# Unraveling the Mechanisms of Wuling Powder Against MASLD by Integrated Metabolomics–Gut Microbiota–Serum Pharmacochemistry

**DOI:** 10.3390/ph19040557

**Published:** 2026-03-31

**Authors:** Huan Yang, Yan-Mei Tang, Shao-Cong Han, Peng-Quan Wang, Yu-Xuan Tao, Hui-Qiong Yang, Min Zhang, Min Li, Jie Yu, Xing-Xin Yang

**Affiliations:** 1 College of Pharmaceutical Science, Yunnan University of Chinese Medicine, 1076 Yuhua Road, Kunming 650500, China; yanghuan_1205@163.com (H.Y.); m18488829594@163.com (Y.-M.T.); axero555@163.com (P.-Q.W.); ttaoyx@163.com (Y.-X.T.); 15752819630@163.com (H.-Q.Y.); 15187474645@163.com (M.Z.); lm08212000@163.com (M.L.); 2 Yunnan Key Laboratory of Southern Medicine Utilization, 1076 Yuhua Road, Kunming 650500, China; hanshaocong_0225@163.com; 3 The First Clinical College, Yunnan University of Chinese Medicine, 1076 Yuhua Road, Kunming 650500, China

**Keywords:** effective components, gut microbiota, metabolic dysfunction-associated steatotic liver disease, metabolomics, Wuling Powder

## Abstract

**Background/Objective:** Metabolic dysfunction-associated steatotic liver disease (MASLD) is a highly prevalent chronic liver disease with no specific therapeutics. Wuling Powder (WLP) is a classic traditional Chinese medicine prescription with therapeutic potential against MASLD, yet its molecular mechanism remains unclear. This study aims to elucidate the mechanism and possible effective substances of WLP in the treatment of MASLD. **Methods:** A rat MASLD model was established via high-fat diet feeding to evaluate WLP’s efficacy. Untargeted metabolomics and 16S rRNA sequencing were used to explore the effects of WLP on metabolism and gut microbiota in vivo. Serum pharmacochemistry combined with metabolomics was used to analyze the key active components and core targets of WLP against MASLD, and molecular docking and cell experiments were used to verify the relationship between them. **Results:** WLP reduced hepatic lipid accumulation and pathological damage, improved lipid levels in blood liver, enhanced antioxidant capacity, and alleviated inflammation in MASLD rats. Mechanistically, WLP regulated 19 metabolic pathways. It also decreased the Firmicutes/Bacteroidota ratio and reduced the abundance of potential pathogenic bacteria (*Romboutsia* and *Turicibacter*). Thirty-one WLP-derived components were identified in serum, 13 of which were key active components for treating MASLD. These components, especially 11-deoxyalisol A and 8β-methoxyatractylenolide I, alleviated hepatic steatosis by downregulating NOS2 and PLA2G2A expression. **Conclusions:** The alleviation of MASLD by WLP was mediated by the regulation of 8 metabolic pathways, alterations in the abundance of *Romboutsia* and *Turicibacter*, and the restoration of 20 metabolite levels, an effect primarily ascribed to 13 distinct pharmacodynamic components derived from WLP.

## 1. Introduction

Metabolic dysfunction-associated steatotic liver disease (MASLD) was formerly termed non-alcoholic fatty liver disease (NAFLD). NAFLD is characterized by hepatic steatosis affecting more than 5% of hepatocytes in the absence of excessive alcohol consumption and other well-defined causes of liver injury. By contrast, MASLD is diagnosed based on hepatic steatosis accompanied by at least one cardiometabolic abnormality, including obesity/abdominal obesity, glycemic disorders/type 2 diabetes mellitus, hypertension, and dyslipidemia [1]. This revised definition more accurately captures the metabolic basis of the disease and its close linkage with multisystem comorbidities. As the most common chronic liver disease globally, MASLD affects up to 38% of the adult population worldwide and represents a leading cause of liver cirrhosis, end-stage liver disease, and primary liver cancer [2]. Currently, lifestyle interventions including low-carbohydrate, low-fat diets and exercise remain the mainstay for weight loss in the management of MASLD. In clinical practice, statins and sodium–glucose cotransporter 2 inhibitors are frequently prescribed for patients with MASLD accompanied by obesity and type 2 diabetes [3]. Nevertheless, statin treatment is associated with an increased risk of rhabdomyolysis, whereas sodium–glucose cotransporter 2 inhibitors may predispose patients to ketoacidosis [4,5]. For metabolic dysfunction-associated steatohepatitis (MASH, the progressive form of MASLD), thyroid hormone receptor β-selective agonists (resmetirom) and glucagon-like peptide-1 receptor agonists (semaglutide) are commonly used, yet both are associated with significant side effects and low efficacy [6,7]. Therefore, the development of safe and effective therapeutic agents for MASLD is of great clinical importance.

The pathogenesis of MASLD is complex, involving multiple factors such as insulin resistance, oxidative stress, abnormal lipid metabolism, genetic variation, and intestinal microbiota dysbiosis [8]. In recent years, traditional Chinese medicine (TCM), characterized by its multi-component, multi-target, and multi-pathway properties, has demonstrated unique advantages in addressing the complex pathogenesis of MASLD [9,10]. Wuling Powder (WLP) was first recorded in Treatise on Febrile Diseases and included in the Chinese Pharmacopoeia (2025 Edition), which is composed of Alismatis Rhizoma (AR), Poria (PA), Polyporus (PS), Atractylodis Macrocephalae Rhizoma (AMR) and Cinnamomi Ramulus (CR) in a 5:3:3:3:2 ratio. WLP has the effects of warming yang to activate qi and strengthening the spleen to dispel dampness and is a very famous dampness-resolving formula in TCM. Modern pharmacological studies have shown that WLP reduces blood lipid levels in hyperlipidemic mice, promotes reverse cholesterol transport and hepatic bile acid metabolism, reshapes the gut microbiota, and restores bile acid homeostasis to alleviate MASLD [11,12]. It also inhibits the release of inflammatory factors to alleviate inflammatory damage and regulate blood glucose and lipid levels in mice with type 2 diabetes [13]. In addition, all single herbs in the prescription can also alleviate MASLD-related symptoms [14,15,16,17]. Clinical studies have found that WLP reduces serum leptin levels in MASLD patients, improves insulin resistance, and normalizes liver function and lipid indices. Moreover, its combination with polyene phosphatidylcholine can enhance therapeutic efficacy [18,19]. Additionally, WLP is indicated for patients with MASLD of the phlegm dampness stasis obstruction type, and it is not suitable for those with heat symptoms. However, the mechanism of action and pharmacodynamic substances of WLP against MASLD remain unclear.

In this study, a MASLD rat model induced by high-fat diet feeding was established to evaluate the efficacy of WLP. Then, untargeted metabolomics and intestinal microbiota analysis were used to investigate the anti-MASLD mechanism of WLP. Furthermore, combined analysis of serum pharmacochemistry and metabolomics was conducted to identify the active components and therapeutic targets of WLP for anti-MASLD. Finally, molecular docking and cell experiments were performed to evaluate the anti-MASLD activity of these active components. This study reveals the anti-MASLD mechanism and in vivo active substances of WLP, thereby providing a scientific basis for its clinical application and market development.

## 2. Results

### 2.1. WLP Reverses the HFD-Induced Pathological Changes in MASLD Rats

**Figure 1:**
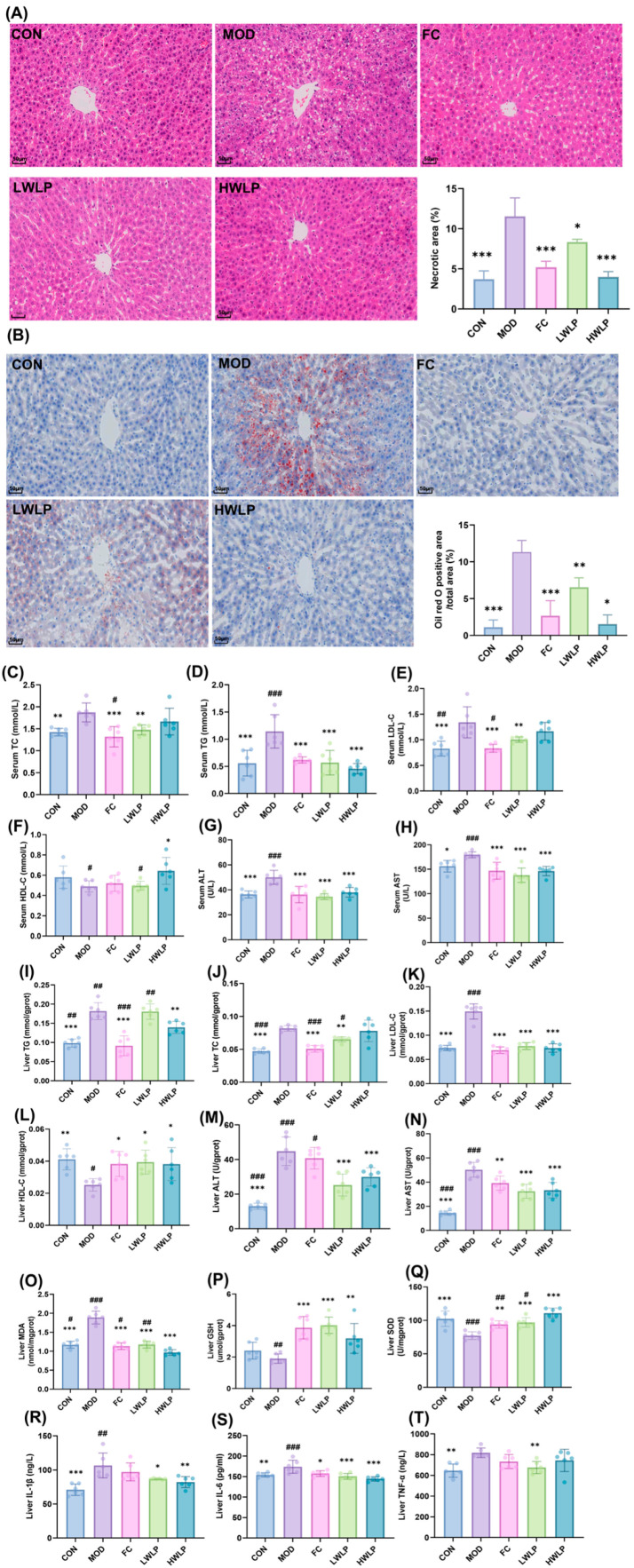
Efficacy of WLP against MASLD. (**A**) HE staining sections, cell nuclei are clearly visualized by blue staining, and the cytoplasm is stained pink. Abnormal fat droplets appear as empty spaces within the cytoplasm (scale bar = 50 μm). (**B**) Oil Red O staining sections, Neutral lipid droplets are stained red, and nuclei are counterstained blue. (scale bar = 50 μm). Quantitative analysis of Oil Red O-positive areas and necrotic areas in HE-stained sections was performed using ImageJ software (version 1.54f). Serum levels of (**C**) TC, (**D**) TG, (**E**) LDL-C, (**F**) HDL-C, (**G**) ALT, and (**H**) AST. Hepatic levels of (**I**) TG, (**J**) TC, (**K**) LDL-C, (**L**) HDL-C, (**M**) ALT, (**N**) AST, (**O**) MDA, (**P**) GSH, (**Q**) SOD, (**R**) IL-1β, (**S**) IL-6, and (**T**) TNF-α. *n* = 6, and the results are expressed as mean ± SD. * *p* < 0.05, ** *p* < 0.01, *** *p* < 0.001 vs. the MOD group. # *p* < 0.05, ## *p* < 0.01, ### *p* < 0.001 vs. the HWLP group. CON, control group. MOD, model group. FC, fenofibrate capsules group. LWLP, low-dose WLP group. HWLP, high-dose WLP group.

HE staining results demonstrated that following 12 weeks of HFD feeding, hepatocytes in the rat liver exhibited disorganized arrangement, accompanied by an increased number of lipid droplet vacuoles. Meanwhile, Oil Red O staining revealed that severe lipid accumulation was observed in the rat liver after 12 weeks of HFD feeding. Treatment with WLP significantly ameliorated the hepatic histomorphology of MASLD rats and alleviated hepatic lipid accumulation and damage (Figure 1A,B). In addition, as shown in Figure 1C–N, following 12 weeks of HFD feeding, the levels of TC, TG, LDL-C, ALT, and AST in the serum and liver of rats were significantly elevated (*p* < 0.05, *p* < 0.01, *p* < 0.001), whereas the level of HDL-C in the liver was significantly reduced (*p* < 0.01). Following intervention with WLP, the serum and hepatic levels of TC, TG, LDL-C, ALT, and AST were significantly decreased (*p* < 0.05, *p* < 0.01, *p* < 0.001), while hepatic HDL-C levels were significantly increased (*p* < 0.05). The HWLP group was significantly superior to the LWLP group in regulating serum HDL-C and liver TC and TG levels (*p* < 0.05, *p* < 0.01), suggesting a dose-dependent effect of WLP treatment. These findings suggest that WLP regulated serum and hepatic lipid levels and ameliorated HFD-induced lipid metabolism disorders.

As shown in Figure 1O–T, following 12 weeks of HFD feeding, the levels of MDA, TNF-α, IL-1β, and IL-6 in the livers of rats were significantly increased (*p* < 0.01, *p* < 0.001), accompanied by a marked decrease in SOD activity (*p* < 0.001). After treatment with WLP, the levels of MDA, TNF-α, IL-1β, and IL-6 were significantly reduced (*p* < 0.05, *p* < 0.01, *p* < 0.001), whereas the activities of GSH and SOD were significantly elevated (*p* < 0.01, *p* < 0.001). The HWLP group was significantly superior to the LWLP group in regulating liver SOD and MDA levels (*p* < 0.05, *p* < 0.01), indicating a dose-dependent effect of WLP treatment. These results indicate that WLP shows promise in alleviating HFD-induced hepatic oxidative stress and inflammatory response in MASLD rats.

### 2.2. WLP Normalizes the Abnormal Metabolic Profiles in Serum, Liver, and Urine of MASLD Rats

**Figure 2:**
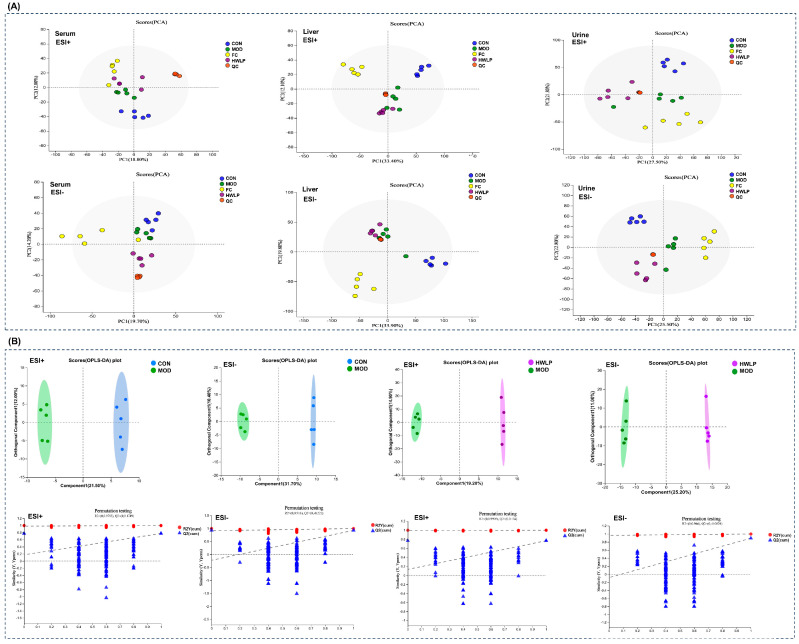

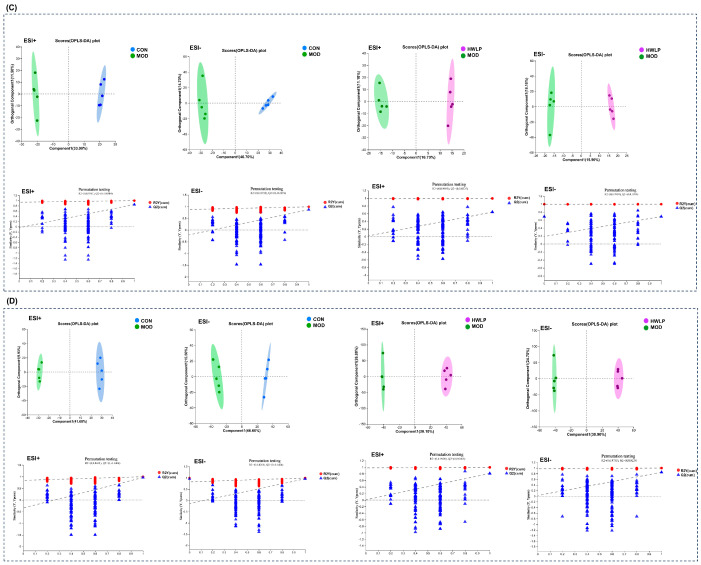
Multivariate analysis of each group. (**A**) PCA score plots. OPLS-DA plot and permutation test plot of (**B**) serum, (**C**) liver, and (**D**) urine. CON, control group. MOD, model group. FC, fenofibrate capsules group. QC, quality control group. ESI+, positive ion mode; ESI−, negative ion mode. In the permutation test plot, the two dashed lines represent the regression lines of R2 and Q2, respectively.

Untargeted metabolomics was employed to investigate the effects of WLP on the metabolic profiles of serum, liver, and urine in MASLD rats. PCA results reveal significant changes in metabolite composition among the CON, MOD, and WLP group (Figure 2A). To further evaluate pairwise differences in metabolic profiles between groups, OPLS-DA was conducted. This analysis showed distinct separation between the CON and MOD groups, as well as between the MOD and WLP groups, confirming that the three groups exhibited distinct metabolic profiles (Figure 2B–D). Additionally, a random permutation test (200 times) was performed for the OPLS-DA model. Model parameters demonstrated that R^2^Y was close to 1 and Q^2^ > 0.5 (Table 1), indicating that the OPLS-DA model had good explanatory and predictive abilities. In addition, the OPLS-DA models for serum ESI+ and liver tissue ESI− exhibited positive Q^2^ intercepts, which suggests a potential risk of overfitting. However, the overall distribution of Q^2^ values after random permutation was lower than that of the original model, suggesting high reliability of the models. Moreover, R^2^ values were greater than 0.9, indicating excellent explanatory power of the models for the data.

**Figure 3:**
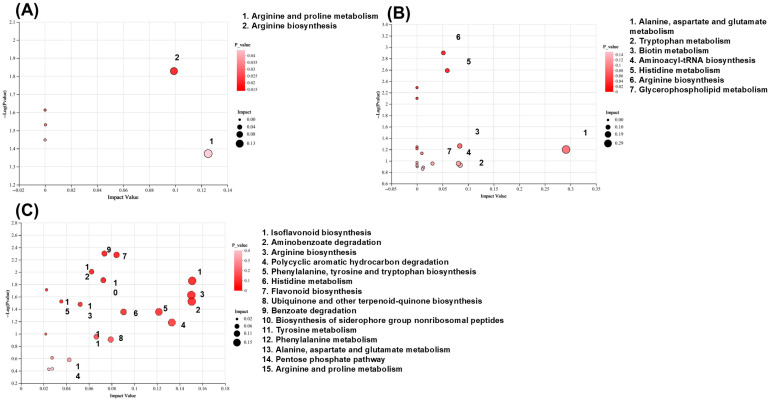
Bubble plot of metabolic pathway enrichment analysis. (**A**) Serum, (**B**) liver, (**C**) urine.

Differential metabolites were screened with the criteria of VIP > 1.0 and *p* < 0.05. After WLP treatment, the levels of 222 differential metabolites in MASLD rats were reversed, including 28 in serum (Table S1), 5 in liver tissues (Table S2), and 192 in urine (Table S3). Subsequently, pathway enrichment analysis was performed for these differential metabolites using the KEGG database. The results indicate that WLP regulated 19 metabolic pathways in MASLD rats, primarily involving amino acid, lipid, cofactor and vitamin metabolism, biosynthesis of other secondary metabolites, biodegradation and metabolism of xenobiotics, and translation-related pathways (Figure 3A–C).

### 2.3. WLP Improves the Gut Microbiota Dysbiosis of MASLD Rats

**Figure 4:**
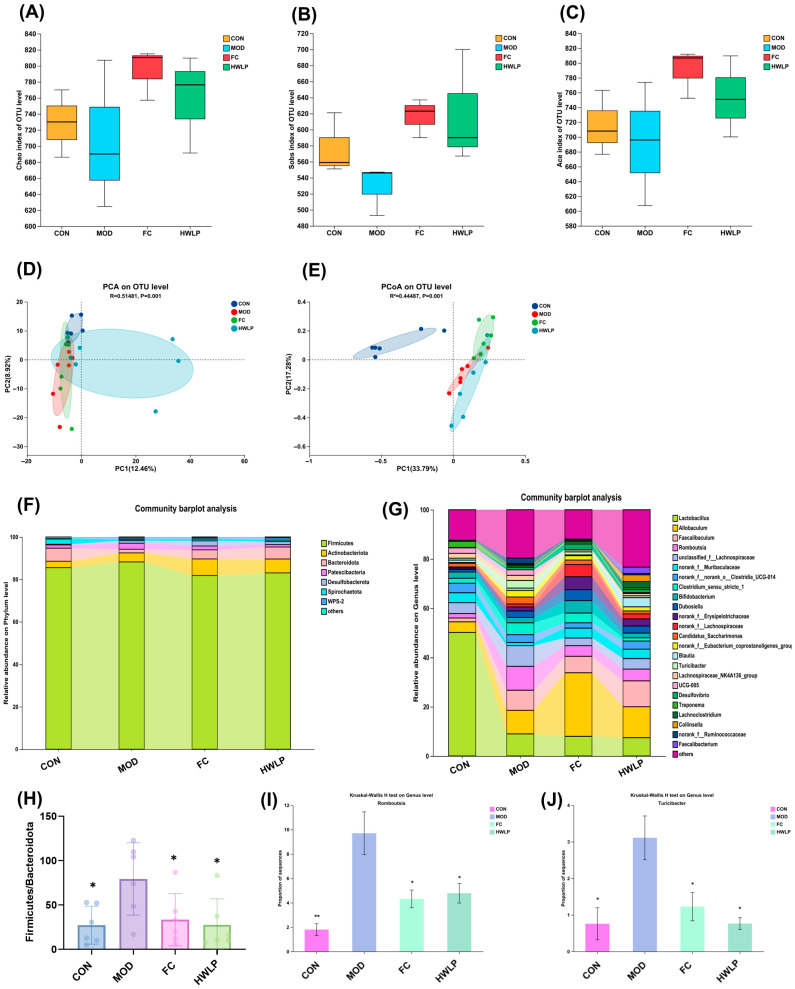
Effect of WLP on gut microbiota in MASLD rats. (**A**) Chao1 index. (**B**) Sobs index. (**C**) Ace index. (**D**) PCA. (**E**) PCoA. (**F**) Species composition at the phylum level of intestinal flora in rats. (**G**) Species composition at the genus level of intestinal flora in rats. (**H**) Firmicutes/Bacteroidota ratio. (**I**) Relative abundance of *Romboutsia*. (**J**) Relative abundance of *Turicibacter*. * *p* < 0.05, ** *p* < 0.01 vs. the MOD group. CON, control group. MOD, model group. FC, fenofibrate capsules group.

Alpha diversity analysis reveals that the Chao, Ace, and Sobs indices exhibited an increasing trend after WLP group treatment, though the difference did not reach statistical significance (Figure 4A–C). Further evaluation of the similarity or dissimilarity in the intestinal flora community structure was conducted using PCA and PCoA. Results showed a clear separation between the WLP and MOD group (Figure 4D,E), indicating that the gut microbiota structure dysbiosis in HFD-induced MASLD rats was ameliorated by WLP. Community bar charts were used to further analyze the species composition of each group across different taxonomic levels. At the phylum level, the intestinal flora of rats was predominantly composed of Firmicutes, Actinobacteriota, Bacteroidota, Patescibacteria, and Desulfobacterota (Figure 4F). Compared with the MOD group, WLP treatment resulted in a decrease in the relative abundance of Firmicutes, an increase in the relative abundance of Bacteroidota, and a significant reduction in the Firmicutes/Bacteroidota ratio *(p* < 0.05) (Figure 4H). At the genus level, WLP significantly decreased the relative abundances of *Romboutsia* and *Turicibacter* in the intestines of MASLD rats (*p* < 0.05) (Figure 4G,I–J). These findings indicate that WLP modified the species composition of intestinal flora in MASLD rats and modulated the homeostasis of the intestinal flora.

### 2.4. Correlation Between the Gut Microbiota, Pathological Indicators, and Differential Metabolites Regulated by WLP

**Figure 5:**
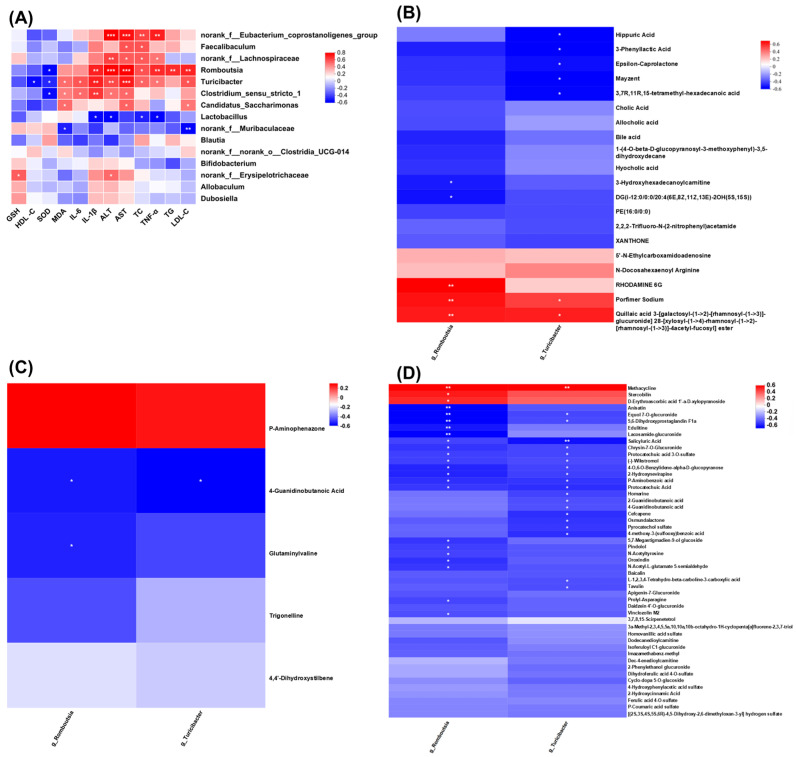
Spearman’s correlation analysis among gut microbiota, pathological indicators, and differential metabolites regulated by WLP. Spearman’s correlation heatmap between gut microbiota at the genus level and (**A**) pathological indicators, (**B**) serum differential metabolites, (**C**) liver differential metabolites, and (**D**) urine differential metabolites. * *p* < 0.05, ** *p* < 0.01, *** *p* < 0.001.

To further explore the potential association between WLP-regulated gut microbiota and MASLD, Spearman correlation analysis was performed between gut microbiota at the genus level and liver pathological indicators. As shown in Figure 5A, *Romboutsia* exhibited a significantly positive correlation with the levels of TC, TG, ALT, AST, LDL-C, IL-1β, and TNF-α (*p* < 0.05, *p* < 0.01, *p* < 0.001), while showing a significantly negative correlation with SOD levels (*p* < 0.05). *Turicibacter* had a significantly positive correlation with the levels of TC, ALT, AST, LDL-C, IL-1β, TNF-α, IL-6, and MDA (*p* < 0.05, *p* < 0.01, *p* < 0.001), and a significantly negative correlation with HDL-C and SOD levels (*p* < 0.05). These results suggest that *Romboutsia* and *Turicibacter* may be the key gut microbiota through which WLP restores gut microbiota homeostasis and alleviates MASLD.

To investigate the association between WLP-regulated endogenous metabolites and gut microbiota, Spearman correlation analysis was conducted between the differential metabolites and the gut microbiota at the genus level reversed by WLP. As shown in Figure 5B, *Romboutsia* showed a significant positive correlation with three serum differential metabolites, quillaic acid 3-[galactosyl-(1->2)-[rhamnosyl-(1->3)]-glucuronide]28-[xylosyl-(1->4)-rhamnosyl-(1->2)-[rhamnosyl-(1->3)]-4acetyl-fucosyl] ester, porfimer sodium, and rhodamine 6g (*p*< 0.01), and a significant negative correlation with DG(i-12:0/0:0/20:4(6E,8Z,11Z,13E)-2OH(5S,15S)) and 3-hydroxyhexadecanoylcarnitine (*p* < 0.05). For *Turicibacter*, it had a significant positive correlation with two serum differential metabolites, quillaic acid 3-[galactosyl-(1->2)-[rhamnosyl-(1->3)]-glucuronide]28-[xylosyl-(1->4)-rhamnosyl-(1->2)-[rhamnosyl-(1->3)]-4acetyl-fucosyl] ester and porfimer sodium (*p* < 0.05), as well as a significant negative correlation with 3,7R,11R,15-tetramethyl-hexadecanoic acid, mayzent, epsilon-caprolactone, hippuric acid, and 3-phenyllactic acid (*p* < 0.05).

As shown in Figure 5C, *Romboutsia* exhibited a significantly negative correlation with the liver differential metabolites 4-guanidinobutanoic acid and glutaminylvaline (*p* < 0.05). *Turicibacter* showed a significantly negative correlation with the liver differential metabolite 4-guanidinobutanoic acid (*p* < 0.05).

As shown in Figure 5D, *Romboutsia* exhibited a significantly positive correlation with the urine differential metabolites methacycline, stercobilin, and D-erythroascorbic acid 1′-α-D-xylopyranoside (*p* < 0.05, *p* < 0.01), while showing a significantly negative correlation with anisatin, equol 7-O-glucuronide, 5,6-dihydroxyprostaglandin F1α, edulitine, lacosamide-glucuronide, salicyluric acid, chrysin-7-O-Glucuronide, protocatechuic acid 3-O-sulfate, (-)-wikstromol, 4-O,6-O-benzylidene-α-D-glucopyranose, 2-hydroxynevirapine, P-aminobenzoic acid, protocatechuic acid, 5,7-megastigmadien-9-ol glucoside, pindolol, N-acetyltyrosine, oroxindin, N-acetyl-L-glutamate 5-semialdehyde, prolyl-Asparagine, and vinclozolin M2 (*p* < 0.05, *p* < 0.01). *Turicibacter* displayed a significantly positive correlation with the urine differential metabolite methacycline (*p* < 0.01), and a significantly negative correlation with equol 7-O-glucuronide, 5,6-dihydroxyprostaglandin F1α, salicyluric acid, chrysin-7-O-glucuronide, protocatechuic acid 3-O-sulfate, (-)-wikstromol, 4-O,6-O-benzylidene-α-D-glucopyranose, 2-hydroxynevirapine, P-aminobenzoic acid, protocatechuic acid, homarine, 2-guanidinobutanoic acid, 4-guanidinobutanoic acid, cefcapene, osmundalactone, pyrocatechol sulfate, 4-methoxy-3-(sulfooxy)benzoic acid, L-1,2,3,4-tetrahydro-β-carboline-3-carboxylic acid, and tavulin (*p* < 0.05, *p* < 0.01).

The above results indicate that WLP ameliorated HFD-induced MASLD by regulating the relative abundance of key gut microbiota and restoring the levels of related endogenous metabolites.

### 2.5. Potential Active Substances for WLP Against MASLD

A total of 31 WLP-derived components were identified in rat serum, with 8 components derived from AR, 2 from PS, 6 from PA, 12 from AMR, and 5 from CR. Among these 31 pharmacodynamic components, there were 12 triterpenoids, 6 sesquiterpenoids, 2 phenolic acids, 5 carboxylic and organic acids, 2 steroids, 1 fatty alcohol, 1 coumarin, 1 phenylpropanoic acid, and 1 glycoside (Table 2).

**Figure 6:**
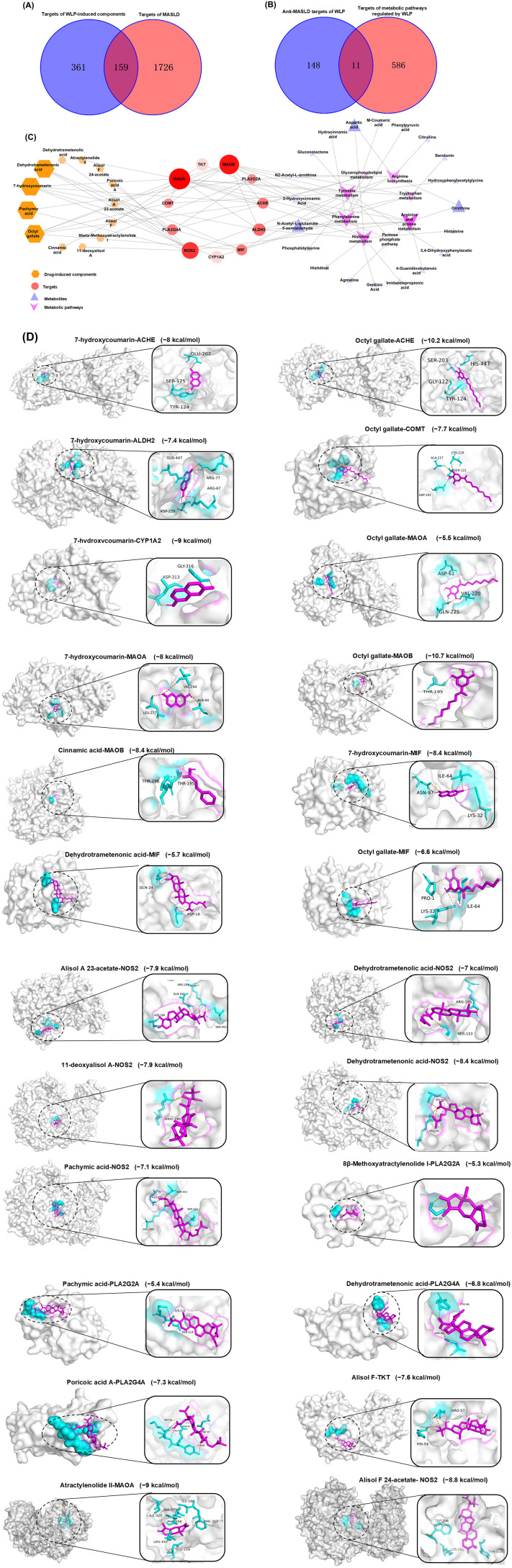
Integrated analysis of WLP-derived components and metabolomics. (**A**) Venn diagram of WLP-derived component targets and MASLD targets. (**B**) Venn diagram of anti-MASLD targets of WLP-derived components and WLP-regulated metabolic pathway targets. (**C**) Component-target-pathway-metabolite network. (**D**) Molecular docking between key components and core targets. The gray indicate crystal structure of target, pink indicate crystal structure of active ingredient and blue indicate crystal structure of amino acid residues.

Subsequently, a network pharmacology analysis was performed on the MASLD targets and WLP-derived component targets. A total of 520 targets of WLP-derived components and 1885 MASLD-related targets were screened out. The number of overlapping targets between the two groups was 159, which are the potential targets of the WLP-derived components for anti-MASLD effect (Figure 6A). A total of 597 relevant targets were obtained from 19 metabolic pathways. These targets were subsequently overlapped with the anti-MASLD targets of the WLP-derived components, resulting in 11 targets, including TKT, NOS2, PLA2G4A, MIF, PLA2G2A, MAOA, MAOB, CYP1A2, ALDH2, ACHE, and COMT (Figure 6B).

By constructing the “component–target–metabolic pathway–metabolite” network via Cytoscape, version 3.10.1 (Figure 6C), it was found that 11 core targets were associated with 13 WLP-derived components. These components may be the key active substances for WLP to alleviate MASLD, including alisol F, alisol F 24-acetate, alisol A 23-acetate, 11-deoxyalisol A, dehydrotrametenolic acid, dehydrotrametenonic acid, pachymic acid, poricoic acid A, 7-hydroxycoumarin, octyl gallate, 8β-methoxyatractylenolide I, atractylenolide II, and cinnamic acid, with their relative contents being 1.31%, 11.04%, 2.29%, 32.14%, 10.97%, 0.62%, 2.04%, 1.34%, 4.60%, 7.71%, 2.44%, 21.99%, and 1.50%, respectively. Additionally, the core targets are involved in 8 metabolic pathways (arginine and proline, phenylalanine, histidine, tyrosine, tryptophan, and glycerophospholipid metabolism, arginine biosynthesis, and pentose phosphate pathway) and affect the levels of 20 metabolites (gluconolactone, N-acetyl-L-glutamate 5-semialdehyde, agmatine, 4-guanidinobutanoic acid, ornithine, citrulline, aspartic acid, N2-acetyl-L-ornithine, phosphatidylserine, gentisic acid, 3,4-dihydroxyphenylacetic acid, hydroxyphenylacetylglycine, phenylpyruvic acid, M-coumaric acid, hydrocinnamic acid, 2-hydroxycinnamic acid, histidinal, imidazolepropionic acid, histamine, and serotonin). Finally, molecular docking between the key active substances and the core targets shows that all binding energies were less than −5.0 kcal/mol (Table 3), suggesting that the 13 key active components derived from WLP exhibit favorable binding ability for the relevant targets, with the binding sites shown in Figure 6D.

### 2.6. Key Active Components Derived from WLP Alleviate FFA-Induced Steatosis in HepG2 Cells

**Figure 7:**
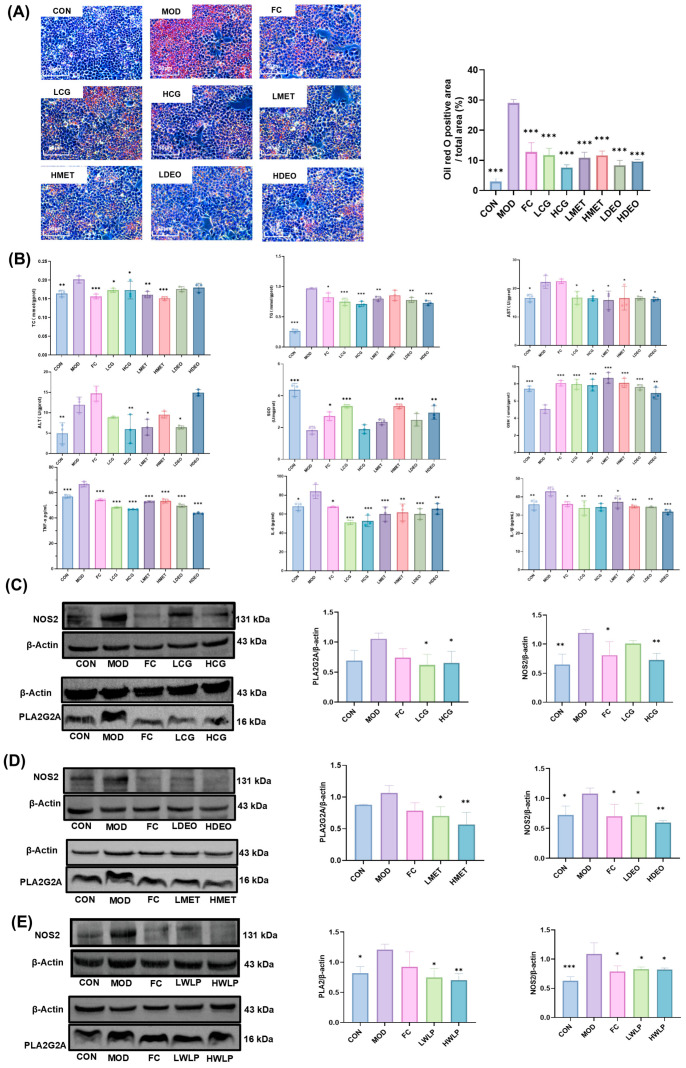
Efficacy of WLP-derived key active components and component groups against hepatic steatosis. (**A**) Oil Red O staining sections, Neutral lipid droplets are stained red, and nuclei are counterstained blue (scale bar = 50 μm); (**B**) the indicators related to lipid metabolism, oxidative stress, and inflammation, including TC, TG, AST, ALT, SOD, GSH, TNF-α, IL-6, and IL-β; (**C**) the expression of NOS2 and PLA2G2A in HepG2 cells after CG treatment; (**D**) the expression of NOS2, PLA2G2A in HepG2 cells after DEO and MET treatment; (**E**) the expression of NOS2 and PLA2G2A in liver tissues after WLP treatment. * *p* < 0.05, ** *p* < 0.01, *** *p* < 0.001 vs. the MOD group. CON, control group. MOD, model group. FC, fenofibrate capsules group. LCG, low-dose WLP-derived component group. HCG, high-dose WLP-derived component group. LMET, low-dose 8β-methoxyatractylenolide I group. HMET, high-dose 8β-Methoxyatractylenolide I group. LDEO, low-dose 11-deoxyalisol A group. HDEO, high-dose 11-deoxyalisol A group.

Among the 13 key active components, only DEO and MET have not been reported to exhibit anti-MASLD activity. Therefore, the anti-MASLD activity of DEO and MET was evaluated using FFA-induced HepG2 cells. Additionally, the in vitro anti-hepatic steatosis capacity of component group composed of 12 key active components (the reference standard of dehydrotrametenonic acid was unavailable and was not mixed into the component group) was assayed. Oil Red O staining results demonstrate that FFA treatment induced lipid accumulation in HepG2 cells, whereas treatment with CG, MET, and DEO significantly reduced intracellular lipid droplet accumulation (Figure 7A). In addition, CG intervention significantly decreased the intracellular levels of TC, TG, ALT, AST, TNF-α, IL-1β, and IL-6 (*p* < 0.05, *p* < 0.01, *p* < 0.001), while the activities of SOD and GSH (*p* < 0.001) were significantly increased; MET significantly decreased the intracellular levels of TC, TG, ALT, AST, TNF-α, IL-1β, and IL-6 (*p* < 0.05, *p* < 0.01, *p* < 0.001), while the levels of SOD and GSH were significantly increased (*p* < 0.001); and DEO intervention significantly lowered the levels of TG, AST, ALT, TNF-α, IL-1β, and IL-6 (*p*< 0.05, *p* < 0.01, *p* < 0.001), while the levels of SOD and GSH were significantly increased (*p* < 0.01, *p* < 0.001) (Figure 7B). These results indicate that CG, MET, and DEO ameliorated FFA-induced lipid accumulation, oxidative stress, and inflammation in HepG2 cells, thereby alleviating steatosis.

The regulatory effects of DEO and MET on the core targets NOS2 and PLA2G2A, respectively, were further verified by Western blot assay. CG treatment significantly down-regulated the expression levels of NOS2 and PLA2G2A in HepG2 cells (*p* < 0.05, *p* < 0.01) (Figure 7C), while DEO and MET intervention significantly down-regulated the expression of NOS2 and PLA2G2A in cells, respectively (*p* < 0.05, *p* < 0.01) (Figure 7D). In addition, after treating MASLD rats with WLP extract, the levels of NOS2 and PLA2G2A in liver tissues were significantly down-regulated (*p* < 0.05, *p* < 0.01) (Figure 7E). These results suggest that DEO and MET improved hepatocellular steatosis by regulating the protein expression of NOS2 and PLA2G2A, respectively.

## 3. Discussion

MASLD is a chronic liver disease characterized by excessive lipid accumulation in hepatocytes induced by metabolic dysfunction as its core pathological feature. Long-term high-calorie diet and lack of exercise have led to a continuous rise in the prevalence of MASLD, which is now the most common chronic liver disease worldwide [20]. Dysregulated lipid metabolism can lead to excessive accumulation of fatty acids in the liver, thereby inducing MASLD, often manifested as abnormal changes in lipid metabolism indicators such as TC, TG, LDL-C, HDL-C, ALT, and AST. Significantly, this study found that WLP alleviated hepatic lipid accumulation and hepatocyte pathological damage in HFD-fed MASLD rats; reduced the levels of TC, TG, LDL-C, ALT, and AST; increased the level of HDL-C; and regulated lipid metabolism disorders. In addition, oxidative stress is a key factor driving the initiation and progression of MASLD. Excessive lipid accumulation in hepatocytes can induce mitochondrial dysfunction, generate large amounts of reactive oxygen species (ROS), trigger lipid peroxidation, and impair hepatic cell membranes and organelles [21]. Furthermore, the progression of MASLD is often accompanied by the occurrence of inflammatory responses. Excessive lipid accumulation in hepatocytes can activate hepatic stellate cells and Kupffer cells to release pro-inflammatory factors, further damaging hepatocytes [22]. In this study, WLP was found to not only increase the hepatic levels of antioxidant molecules (GSH, SOD) and reduce MDA levels but also markedly suppress the expression of pro-inflammatory cytokines (TNF-α, IL-1β, and IL-6). These findings indicate that WLP ameliorated abnormal hepatic oxidative stress and inflammatory responses to mitigate HFD-induced MASLD.

Metabolomics technology enables the reflection of an organism’s physiological and pathological states through detecting dynamic changes in small-molecule metabolites within the organism. In recent years, this technology has played a crucial role in research on the modernization of TCM [23]. In this study, it was found that after intervention with WLP, 28, 5, and 192 potential differential metabolites in serum, liver, and urine could be reversed respectively, and 19 metabolic pathways were mainly regulated. Aberrant amino acid metabolism is a key driver of the pathogenesis of MASLD. Emerging evidence indicates that aberrant amino acid metabolism can trigger oxidative stress, thereby further exacerbating hepatocellular damage [24]. Citrulline, an amino acid with potential hepatoprotective effects, has been confirmed to significantly ameliorate hepatic steatosis [25]. Its precursor, ornithine, collaborates with arginine and proline in the body’s urea cycle, dynamically regulating arginine biosynthesis and proline metabolic homeostasis [26]. Additionally, the endogenous metabolites of arginine, 4-guanidinobutanoic acid and agmatine can exert hepatoprotective effects by modulating mitochondrial function and fatty acid oxidation [27,28]. In the present study, intervention with WLP was found to increase the levels of argininic acid, citrulline, ornithine, 4-guanidinobutanoic acid, and agmatine. Collectively, these findings indicate that WLP alleviates MASLD by restoring the imbalance in amino acid metabolism.

Dysregulation of lipid metabolic homeostasis is the core pathological mechanism underlying the development of MASLD. As the most abundant structural phospholipids in the body, phosphatidylcholine (PC) and phosphatidylethanolamine (PE) play a crucial role in maintaining the dynamic balance of hepatocellular lipids by regulating the glycerophospholipid metabolic pathway [29]. Specifically, studies have shown that PC participates in the assembly of TG and apolipoprotein B, promotes the synthesis of very-low-density lipoprotein (VLDL), and facilitates its conversion to HDL-C, thereby reducing hepatic TG accumulation [30]. In contrast, PE (16:0/0:0), a key precursor for PC synthesis, can regulate the homeostasis of energy metabolism by stabilizing mitochondrial membrane protein complexes [31]. Additionally, metabolites including bile acids, allocholic acid, and hyocholic acid are well-documented to regulate hepatic lipid metabolism [32,33]. Significantly, this study found that WLP intervention elevated the levels of PE(16:0/0:0), bile acids, allocholic acid, and hyocholic acid. These findings suggest that WLP alleviates MASLD by regulating hepatic lipid metabolism disorders.

Excessive carbohydrate intake is a key pathogenic factor in MASLD. Studies have shown that high carbohydrate load can promote de novo fatty acid synthesis, leading to abnormal accumulation of TG. It also produces excessive ROS and aggravates hepatocyte injury [34]. Gluconolactone, as a biosynthetic precursor of D-gluconate, plays a key role in glycolysis and the pentose phosphate pathway. Its downregulation would impair hepatic glucose metabolism function. In this study, it was found that the level of gluconolactone significantly increased after intervention with WLP.

Furthermore, cofactor and vitamin metabolism are important regulators in maintaining hepatic metabolic homeostasis. As a precursor substance for the biosynthesis of ubiquinones and terpenoquinones, 4-hydroxybenzoic acid can alleviate oxidative stress-induced hepatocellular injury by enhancing mitochondrial function [35,36]. Protocatechuic acid, a key intermediate in the synthesis of non-ribosomal peptide siderophores, can ameliorate the hepatic inflammatory microenvironment by inhibiting the release of pro-inflammatory factors [37]. WLP intervention significantly elevated the levels of 4-hydroxybenzoic acid and protocatechuic acid. Additionally, aspartic acid can reduce liver injury by modulating inflammatory cytokine levels. Its precursor, prolyl-asparagine, was significantly increased after WLP intervention. Finally, the biosynthesis of other secondary metabolites, as well as the biodegradation and metabolism of xenobiotics, play crucial roles in the pathological regulation of MASLD. Isoflavonoids such as daidzein and biochanin A can counteract MASLD by regulating hepatic lipogenesis [38,39]. Moreover, 4-hydroxybenzoic acid and protocatechuic acid, as degradation products of benzoic acid, reduce hepatic accumulation of lipotoxic metabolic intermediates by regulating the exogenous substance detoxification process mediated by cytochrome P450 enzymes [40]. WLP intervention increased the levels of daidzein, biochanin A, pinocembrin, 4-hydroxybenzoic acid, and protocatechuic acid. These findings suggest that WLP alleviated MASLD by modulating the levels of various endogenous metabolites and influencing multiple metabolic pathways.

Both the liver and intestine are important components of the digestive system, connected by the portal vein and interacting with each other. The intestine harbors a large number of intestinal flora; dysbiosis of intestinal flora can lead to disorders of bile acid metabolism, endotoxin translocation, and disturbances in metabolites, thereby promoting the progression of MASLD [41]. In this study, it was found that WLP significantly reduced the Firmicutes/Bacteroidota ratio and reduced the relative abundance of *Romboutsia* and *Turicibacter* in the intestines of MASLD rats. Furthermore, increased abundance of *Romboutsia* and *Turicibacter* has been reported in patients with MASLD [42,43]. Significantly, *Romboutsia* and *Turicibacter* showed a significant positive correlation with pathological indicators in the liver, such as TC, TG, ALT, AST, LDL-C, IL-1β, TNF-α, IL-6, and MDA.

Changes in the structure of gut microbiota can affect the levels of bacterial metabolites, thereby influencing MASLD. In this study, the results of correlation analysis between differential metabolites and key flora showed that 4-guanidinobutanoic acid, equol 7-O-glucuronide, (-)-wikstromol, and protocatechuic acid were significantly negatively correlated with the potential pathogenic bacteria *Romboutsia* and *Turicibacter*. Tercobilin was significantly positively correlated with *Romboutsia*, while hippuric acid and 3-phenyllactic acid were significantly negatively correlated with *Turicibacter*. Existing studies have found that 4-guanidinobutanoic acid, as an arginine metabolite, can prevent and treat MASLD by regulating mitochondrial dysfunction. Equol 7-O-glucuronide is a substrate for human hepatic β-glucuronidase and regulates carbohydrate metabolism in the body [44]. (-)-wikstromol, protocatechuic acid, and 3-phenyllactic acid, as intestinal metabolites, have been shown to alleviate oxidative stress, regulate intestinal microbial composition, and possess the potential to treat MASLD [45,46,47]. Additionally, increased levels of hippuric acid are negatively correlated with the prevalence of metabolic syndrome [48]. Stercobilin, on the other hand, is the terminal metabolite produced by the reduction in bilirubin by intestinal flora, and its increase can induce low-grade chronic inflammation in ob/ob mice [49]. The above results suggest that WLP may improve MASLD by regulating metabolite levels through key gut microbiota. Additionally, the differential metabolites significantly associated with gut microbiota also include porfimer sodium, rhodamine 6g, methacycline, D-erythroascorbic acid 1′-a-D-xylopyranoside, anisatin, 5,6-dihydroxyprostaglandin F1a, edulitine, lacosamide-glucuronide, salicyluric acid, chrysin-7-O-glucuronide, protocatechuic acid 3-O-sulfate, 4-O,6-O-benzylidene-alpha-D-glucopyranose, 2-hydroxynevirapine, P-aminobenzoic acid, 5,7-megastigmadien-9-ol glucoside, pindolol, oroxindin, N-acetyl-L-glutamate 5-semialdehyde, prolyl-asparagine, vinclozolin M2, glutaminylvaline, DG(i-12:0/0:0/20:4(6E,8Z,11Z,13E)-2OH(5S,15S)), 3-hydroxyhexadecanoylcarnitine, 3,7R,11R,15-tetramethyl-hexadecanoic acid, mayzent, epsilon-caprolactone, homarine, 2-guanidinobutanoic acid, cefcapene, osmundalactone, pyrocatechol sulfate, 4-methoxy-3-(sulfooxy)benzoic acid, L-1,2,3,4-Tetrahydro-beta-carboline-3-carboxylic acid, and tavulin. None of these differential metabolites have been reported to be associated with the pathogenesis of MASLD, which warrants further investigation.

Serum pharmacochemistry technology of TCM is a method that uses modern scientific technologies and approaches to identify the migrated components in serum after oral administration of TCM. In this study, a total of 31 WLP-derived components were identified in the serum of MASLD rats. Integrated analysis of serum pharmacochemistry combined with metabolomics identified 13 key WLP-derived components, which acted on 11 targets, regulating 8 metabolic pathways and 20 metabolites to exert anti-MASLD effects. Among them, alisol F, alisol F 24-acetate, pachymic acid, poricoic acid A, and octyl gallate possess anti-inflammatory activity and can alleviate MASLD [50,51,52,53,54,55,56,57,58]. Alisol A 23-acetate can improve abnormal lipid metabolism and oxidative stress in hepatocytes [59]. Atractylenolide II and dehydrotrametenolic acid can improve insulin resistance [60]. 7-hydroxycoumarin alleviates liver injury by activating Nrf2, and cinnamic acid improves MASLD by inhibiting hepatic lipogenesis and promoting fatty acid oxidation [61,62]. Moreover, we found that DEO and MET could alleviate hepatic steatosis. The component group composed of 12 key active components also prevented MASLD. This suggests that these 13 components are the key pharmacodynamic substances of WLP against MASLD by regulating 11 core targets, 8 metabolic pathways and 20 metabolites.

Additionally, DEO and MET have not been previously reported for treating MASLD. Further combined analysis revealed that DEO targets NOS2, while MET act on PLA2G2A. NOS2 (iNOS) is a key enzyme that catalyzes the production of NO from L-arginine. Excessive activation of NOS2 leads to overproduction of NO, which impairs hepatic mitochondrial function via nitrative stress, inhibits fatty acid oxidation, and results in massive accumulation of lipids such as triglycerides in the liver. Meanwhile, NO modifies insulin receptor substrates, suppresses insulin signaling, and exacerbates insulin resistance [63]. Furthermore, excess NO derived from NOS2 stimulates the secretion of pro inflammatory cytokines including TNF α, IL 1β, and IL 6, recruits macrophages and T cells into the liver, aggravates hepatic inflammation and injury, and drives the transition from simple steatosis to MASH [64]. Clinical studies demonstrate that hepatic NOS2 expression is markedly elevated in MAFLD patients compared with healthy controls, and is further upregulated in MASH and liver fibrosis relative to simple steatosis. PLA2G2A is a member of the phospholipase A2 family and catalyzes the hydrolysis of phosphatidylcholine, releasing free fatty acids and lysophospholipids, which exacerbate hepatic steatosis. Furthermore, upregulation of PLA2G2A promotes arachidonic acid production, which is further metabolized into inflammatory mediators such as leukotrienes. Previous studies have shown that PLA2G2A expression is increased in rats with liver fibrosis [65]. Thus, NOS2 and PLA2G2A may serve as promising biomarkers for evaluating disease severity and predicting prognosis in MASLD. This study innovatively found that DEO and MET downregulated the expression levels of NOS2 and PLA2G2A in HepG2 cells, respectively, thereby reducing lipid accumulation, oxidative stress damage, and inflammation. These results further describe that the 13 key components may act on their 11 core targets to relieve MASLD.

WLP is a very famous dampness-resolving formula that functions to invigorate the spleen and eliminate dampness. Notably, invigorating the spleen and resolving dampness represents an effective strategy for the prevention and treatment of MASLD; WLP can effectively treat the pathological mechanism of MASLD. In the present study, WLP significantly reduced the levels of TC, TG, and LDL-C in both serum and hepatic tissue and inhibited the release of inflammatory factors, demonstrating its dual functions of lipid lowering and anti-inflammatory effects. Further mechanistic investigations revealed that WLP exerted its therapeutic effects by modulating gut microbiota and 19 metabolic pathways, which were mainly attributed to its 13 medicinal components. Given the current clinical lack of drugs that can simultaneously regulate multiple lipid parameters including TC, TG, and LDL-C and inhibit inflammation, as well as agents capable of targeting multiple targets, gut microbiota, and diverse metabolic pathways for fatty liver disease, WLP shows great promise as a potential therapeutic agent for MASLD.

Currently, the efficacy of WLP in the treatment of MASLD has been scientifically evaluated in clinical settings. However, studies on the application of WLP in the management of MASLD complications remain limited, and relevant research regarding its adverse reactions in clinical practice is also scarce. In this study, although WLP exerted a dose-dependent effect against MASLD, only the high-dose group was included in mechanistic experiments. Further studies are therefore warranted to investigate the regulatory effects of low-dose WLP on the gut microbiota and differential metabolites, and to explore the dose-dependent manner of WLP. The precise causal relationships among molecules, gut microbiota, and metabolic pathways also deserve further validation. In addition, we established a high-fat diet-induced MASLD animal model. Whether WLP also exerts therapeutic effects on other types of fatty liver disease, such as malnutrition-induced fatty liver and toxin-induced fatty liver, remains unclear. Given the increasing prevalence of obesity among populations with long-term high-fat diets, future studies may perform clinical trials in such cohorts. Clinical trials can further validate the mechanisms by which WLP modulates gut microbiota and endogenous differential metabolites.

## 4. Materials and Methods

### 4.1. Reagents and Chemicals

Laboratory animal diet was purchased from SPF (Beijing) Biotechnology Co., Ltd. (Beijing, China). Fenofibrate capsules (FC) were purchased from French Libofoni Pharmaceutical Biological Technology Co., Ltd. (Paris, France). Commercial kits for the determination of total cholesterol (TC), triglyceride (TG), low-density lipoprotein cholesterol (LDL-C), high-density lipoprotein cholesterol (HDL-C), alanine transaminase (ALT), aspartate transaminase (AST), malondialdehyde (MDA), superoxide dismutase (SOD), and glutathione (GSH) were purchased from Nanjing Jiancheng Bioengineering Institute (Nanjing, China). Interleukin-6 (IL-6), interleukin-1β (IL-1β), and tumor necrosis factor-alpha (TNF-α) ELISA Kit were obtained from Jiangsu Meimian Industrial Co., Ltd. (Yancheng, China), and 4% Paraformaldehyde fix solution, hematoxylin and eosin (HE) stain kit, and Oil red O dye were purchased from Wuhan Servicebio Technology Co., Ltd. (Wuhan, China). The HepG2 human hepatocellular cell was purchased from the Cell Resource Center, Institute of Basic Medical Sciences, Chinese Academy of Medical Sciences. (Beijing, China). Dulbecco’s modified eagle medium (DMEN; No.6124477) was purchased from Grand Island Biological Company Co., Ltd. (Grand Island, NY, USA). Sodium oleate (No. S817542) was purchased from Shanghai Macklin Biochemical Co., Ltd. (Shanghai, China). Sodium palmitate (No. P9767) was purchased from Sigma-Aldrich (St. Louis, MO, USA). RIPA Lysis Buffer (No. 240007014) was purchased from Solarbio Co., Ltd. (Beijing, China). Tris Buffered Saline Tween (TBST; No. 240011012) was purchased from Solarbio Co., Ltd. (Beijing, China). Ultrasensitive ECL Detection Kit (No. 230718E01-02) was obtained from UElandy Co., Ltd. (Suzhou, China). PageRuler™ Plus Prestained Protein Ladder, 10 to 180 kDa (26616) was purchased from Thermo Fisher Scientific Co., Ltd. (Waltham, MA, USA). β-actin (No. 20536-1-AP) was purchased from Proteintech Group, Inc. (Wuhan, China). Goat Anti-Rabbit IgG(H + L) (No. Bs-0295G-HRP) and Anti-NOS2 (No. Bs-0162R) were purchased from Bioss Biotechnology Co., Ltd. (Beijing, China). Anti-PLA2G2A (No. TU721565S) was purchased from Abmart Inc. (Shanghai, China). Alisol F 24-acetate (No. TC0328-231129), octyl gallate (No. TC4627-240429), atractylenolide II (No. TC0526-240325), and cinnamic acid (No. TC0822-230522) were purchased from Sichuan Jingcui Tiancheng Pharmaceutical Technology Co., Ltd. (Chendu, China). Dehydrotrametenolic acid (No. QC90577), pachymic acid (No. QC99590), alisol A 23-acetate (No. QC92544), 11-deoxyalisol A (No. QC95661), poricoic acid A (No. QC92838), 7-hydroxycoumarin (No. QC97503), 8β-Methoxyatracty lenolide I (No. QC89314), and alisol F (No. QC92402) were purchased from Shanghai Qincheng Biotechnology Co., Ltd. (Shanghai, China). All the other reagents were of analytical grade or higher. The AR, PA, PS, AMR and CR samples were purchased from Kunming Luosiwan Chinese Medicinal Materials Market (Kunming, China) and authenticated by Professor Xingxin Yang at the Yunnan University of Chinese Medicine.

### 4.2. Preparation of WLP Extract

The powders of AR, PA, PS, AMR and CR twig were weighed and mixed at a 5:3:3:3:2 ratio, and the mixture was then decocted twice with a 10-fold volume of water for 1 h each time. Impurities were removed by filtration, and the filtrate was retained, concentrated and freeze-dried to produce a freeze-dried powder of WLP aqueous extract with a yield of 14%. The quantitative determination of cinnamaldehyde in WLP was conducted, and its content was found to be 0.36 mg/g (Figure S1, details are provided in the Supplementary Material).

### 4.3. Evaluation of WLP Against MASLD

#### 4.3.1. Animals and Experimental Design

Thirty specific pathogen-free male Sprague-Dawley rats (aged 8 weeks, 180–200 g) were purchased from Beijing Speifu Biotechnology Co., Ltd. (Beijing, China; license No. SCXK, 2019-0010) and kept at 22 ± 2 °C with 60% relative humidity and 12 h light/dark cycle. After one week of adaptive feeding, 30 rats were randomly divided into the following five groups (*n* = 6): control group (CON, fed a normal basal diet), model group (MOD, fed a HFD for 12 weeks to induce MASLD), fenofibrate capsules group (FC, fed with HFD and intragastric administered 21 mg/kg/d of FC for 12 weeks), low-dose WLP group (LWLP, fed with HFD and intragastric administered 5.04 g/kg/d of WLP for 12 weeks), and high-dose WLP group (HWLP, fed with HFD and intragastric administered 20.16 g/kg/d of WLP for 12 weeks). The dosage of WLP was selected based on the maximum effective and safe doses from previous animal experiments and clinical studies [66,67]. The body weight of rats was monitored and recorded weekly. At the 12th week, urine was collected within 24 h in metabolic cages and stored at −80 °C. At the end of the experiment, the rats were anesthetized with pentobarbital sodium, and blood samples were collected from the abdominal aorta and centrifuged (3500 rpm, 15 min, 4 °C) to obtain serum. In addition, the liver and colon contents of the rats were collected for further analysis. All experimental animal manipulations in this study were performed in accordance with the ethical guidelines of the Experimental Animal Ethics Committee of Yunnan University of Chinese Medicine (Approval No. R-062022135).

#### 4.3.2. Histological Analysis

Liver tissues were fixed in 4% paraformaldehyde for 24 h, washed with PBS, dehydrated in gradient ethanol, embedded in paraffin, and sliced into sections, and after hematoxylin-eosin (HE) staining, the cellular morphological changes in rat liver tissue were observed under a light microscope (Olympus Corporation, Tokyo, Japan). Furthermore, liver tissues were embedded in OTC embedding medium, frozen at −20 °C for 30 min, and sectioned at a thickness of 8 μm. The sections were rinsed with 60% isopropanol, stained with Oil Red O staining solution, and subsequently observed under an optical microscope (Olympus Corporation, Tokyo, Japan) to assess lipid accumulation in rat liver tissues. Oil Red O-positive areas and necrotic areas in HE-stained sections were quantified using ImageJ software.

#### 4.3.3. Detection of Biochemical Indicators and Cytokines

The levels of TC, TG, HDL-C, LDL-C, AST, ALT, SOD, MDA, GSH, TNF-α, IL-6, and IL-1β were detected by biochemical kits according to the manufacturer’s instructions and protocols. Data were acquired using a SpectraMax Plus 384 microplate reader (Molecular Devices, Sunnyvale, CA, USA).

### 4.4. Metabolomic Analysis

#### 4.4.1. Sample Preparation

Serum samples of 100 μL were collected from each of the CON, MOD, FC, and HWLP groups, and each sample was mixed with 400 μL extraction solution (acetonitrile: methanol = 1:1). After vortex for 30 s and ultrasonic extraction for 30 min (4 °C, 40 KHz), the samples were centrifuged (12,000 rpm, 15 min, 4 °C). The supernatants were collected, dried under nitrogen, and reconstituted with 100 µL of reconstitution solution (acetonitrile: water = 1:1) prior to analysis. Urine samples were processed in the same manner.

An amount of 50 mg of liver samples from the CON, MOD, FC, and HWLP groups was weighed respectively, and each sample was mixed with 400 μL of extraction solution (methanol: water = 4:1). The samples were ground in a tissue grinder for 6 min (−10 °C, 50 Hz), ultrasonicated for 30 min (5 °C, 40 kHz), and then centrifuged (12,000 rpm, 15 min, 4 °C). The supernatant was collected, dried with nitrogen gas, redissolved in 100 µL of a solvent mixture (acetonitrile:water = 1:1), and then analyzed.

All samples were mixed in equal volume to prepare quality control (QC) samples. During the detection process, one QC sample was run every 5 test samples to evaluate the repeatability of the analytical procedure.

#### 4.4.2. LC-MS/MS Analyses

LC-MS/MS analyses were executed on an Thermo UHPLC-Q Exactive system (Thermo Scientific, Waltham, MA, USA) and a ACQUITYHSS T3 column (1.8 μm, 100 mm × 2.1 mm, Waters, Waltham, MA, USA). The mobile phase comprised (A) 0.1% formic acid in water:acetonitrile (95:5, *v*/*v*) and (B) 0.1% formic acid inacetonitrile: isopropanol:water (47.5:47.5:5, *v*/*v*). The following elution order was adopted: 0–3 min, 5% B→20% B; 3–4.5 min, 20% B→35% B; 4.5–5 min, 35% B→100% B; 5–6.3 min, 100% B; 6.3–6.4 min, 100% B→0% B; 6.4–8 min, 0% B. The sample injection volume was 3 μL, with a flow rate of 0.4 mL/min. Mass spectral signals of the samples were acquired in both positive and negative ion scanning modes using a Thermo UHPLC-Q Exactive Mass Spectrometer equipped with an electrospray ionization (ESI) source, and the scan mass range was 70–1050 *m*/*z*. The spray voltage was 3500 V for the positive ion mode and 2800 V for the negative ion mode. The sheath gas flow rate was 40 psi and the auxiliary gas flow rate was 10 psi. The collision energy was set to a cyclic collision energy of 20–40–60 V.

#### 4.4.3. Data Processing

The raw LC-MS/MS data were preprocessed using Progenesis QI software (Waters Corporation, Milford, MA, USA, version v3.0). In addition, the metabolites were identified using public databases HMDB (accessed on 25 April 2023, https://hmdb.ca/), Metlin (accessed on 25 April 2023, https://metlin.scripps.edu/auth-login.html) and Majorbio. Principal component analysis (PCA) and orthogonal least partial squares discriminant analysis (OPLS-DA) were performed using the R package “ropls” (Version 1.6.2). Significant differential metabolites were identified based on variable importance in projection (VIP) values from the OPLS-DA model and raw *p*-values from Student’s *t*-test; to control false positives caused by multiple testing, the raw *p*-values were corrected using the Benjamini–Hochberg (BH) method, with VIP > 1 and FDR-adjusted *p* < 0.05 as the screening criteria. Significant differential metabolites were identified based on the variable weight value (VIP) obtained from the OPLS-DA model and Student’s *t*-test *p* value (VIP > 1, *p* < 0.05). KEGG (accessed on 25 April 2023, https://www.kegg.jp/kegg/pathway.html) and MetaboAnalyst6.0 (accessed on 25 April 2023, https://www.metaboanalyst.ca/) databases were used to further enrich and analyze the pathways involved in differential metabolites.

### 4.5. 16S rRNA Gene Sequencing Analysis

Total genomic DNA was extracted from rat colonic content samples of the CON, MOD, and HWLP groups using the PF Mag-Bind Stool DNA Kit (Omega, Norcross, GA, USA). The concentration and purity of the DNA were determined by 1% agarose gel electrophoresis. The V3–V4 region of the 16S rRNA gene was amplified by polymerase chain reaction (PCR) using primers [338F (5′-ACTCCTACGGGAGGCAGCAG-3′) and 806R (5′-GGACTACHVGGGTWTCTAAT-3′)]. The PCR product was extracted from 2% agarose gel and purified. Purified PCR products were subjected to library construction using the NEXTFLEX Rapid DNA-Seq Kit (Bioo Scientific, Austin, TX, USA) and finally sequenced on the Illumina PE300/PE250 platform (Illumina, San Diego, CA, USA). Data were clustered into different Operational Taxonomic Units (OTUs) with a 97% similarity threshold using UPARSE software (accessed on 24 April 2023, https://www.drive5.com/uparse/index.html, version 11) for cluster analysis. For each OTU, the sequence with the highest abundance was chosen as the representative sequence. After manual filtration of the OTU table, the 16S rRNA gene sequences of each sample were rarefied to 20,000 reads to reduce the impact of sequencing depth on alpha and beta diversity analyses. Taxonomic annotation of OTUs was performed using the RDP Classifier version 2.2 with a confidence threshold of 70%. The richness and diversity of microorganisms were evaluated using Alpha diversity analysis, such as Chao 1, Ace, and Sobs indices. Differences in microbial community structures among sample groups were examined through Beta diversity analysis, including PCA and Principal coordinate analysis (PCoA).

### 4.6. Correlation Analysis Between Gut Microbiota, Pathological Indicators, and Differential Metabolites

Based on Spearman correlation analysis (|r| > 0.6, *p* < 0.05), the correlations between gut microbiota, pathological indicators, and differential metabolites were analyzed.

### 4.7. Identification of WLP-Derived Components in Serum from MASLD Rats

#### 4.7.1. Sample Processing

A 1.2 mL amount of MOD, HWLP serum was mixed with 3.6 mL of acetonitrile, vortexed for 30 s, allowed to stand for 20 min at 4 °C, and centrifuged (12,000 rpm, 15 min, 4 °C). The supernatant was collected, gently blow-dried under nitrogen gas, then redissolved in 200 μL of an acetonitrile–water mixture (50:50, *v*/*v*) and filtered through a 0.22 μm microporous filter membrane for LC-MS analysis.

#### 4.7.2. LC-MS/MS Analysis

Agilent 1290 infinity II UPLC system (Agilent Technologies, Santa Clara, CA, USA) was used for the analysis. Elution was performed using the Waters Acquity UPLC HSS T3 column (100 mm × 2.1 mm, 1.8 μm, column temperature 30 ◦C, Waters, Milford, MA, USA) at a constant flow rate of 0.2 mL/min. The injection volume was 5 μL. The mobile phase was acetonitrile (A) with 0.1% formic acid water (B). Gradient elution conditions were as follows: 0–5.0 min, 5% A; 5.0–10.0 min, 20% A; 10.0–15.0 min, 40% A; 15.0–20.0 min, 50% A; 20.0–25.0 min, 60% A; 25.0–30.0 min, 70% A; 30. 0–35.0 min, 80% A; 35.0–40.0 min, 90% A; 40.0–41.0 min, 100% A; and 41.0 min, 5% A.

Mass spectrometry was performed by the Agilent G6545 Q/TOF mass spectrometer equipped with ESI (Agilent Technologies, Santa Clara, CA, USA). The positive and negative ion scanning was used to collect the sample quality spectrum information, and the scan mass range was 50–1700 *m*/*z*. The spray voltage was 3.5 kV for the positive ion mode and 2.8 kV for the negative ion mode. The drying temperature was set at 320 °C and the flow rate was 8 L/min. The sheath temperature was 350 °C and the flow rate was 11 L/min. The collision energies were set to 35 and 40 eV.

#### 4.7.3. Data Analysis

Qualitative Analysis B.06.00 software was used to process the UPLC-Q/TOF MS data. First, the mass spectral information of the WLP treatment group was compared with that of the MOD group to eliminate interference from endogenous substances in the serum. Subsequently, the molecular weight, MS/MS data, and relative retention time of AR, PA, PS, AMR, and CR components in the reported studies were used to identify the structures of drug-derived compounds in the serum samples of rats.

### 4.8. Integrated Analysis of Drug-Derived Components and Metabolomics

The targets of WLP-derived components in serum were predicted by PubChem (accessed on 17 April 2024, https://pubchem.ncbi.nlm.nih.gov/) and SwissTargetPrediction (accessed on 17 April 2024, https://swisstargetprediction.ch/). MASLD-related genes were found by GeneCards (accessed on 17 April 2024, https://www.genecards.org/) and OMIM Gene Map (accessed on 17 April 2024, https://www.omim.org/) databases. In addition, related targets of WLP in regulating metabolic pathways were obtained via KEGG (accessed on 22 April 2024, https://www.genome.jp/kegg/pathway.html) website. Venny 2.1.0 (accessed on 22 April 2024, https://bioinfogp.cnb.csic.es/tools/venny/) software was used to obtain the overlapping targets of WLP-derived components, MASLD, and metabolic pathways. Finally, cytoscape was used to construct the active ingredient–target–metabolic pathway–metabolite network to determine the key active components, targets and pathways of WLP alleviating MASLD.

### 4.9. Molecular Docking of WLP-Derived Key Active Components with Its Core Targets

The structures of key active components from WLP were downloaded from the Pubchem (accessed on 22 April 2024, http://pubchem.ncbi.nlm.nih.gov/) website and converted to PDB format using Open Babel 3.0.1 software. Overlapping proteins were searched in the Uniprot (accessed on 22 April 2024, https://www.uniprot.org/uniprotkb) database and their crystal structures were downloaded in the Protein Data Bank (accessed on 22 April 2024, https://www.rcsb.org/). The receptor proteins were treated with Pymol software (version 2.5) to remove water molecules, demetal ions and hydrogenation using AutoDockTools (version 1.5.7). Finally, AutoDock Vina was used to dock the receptor proteins with the active components, the binding energy was calculated, and the docking results were visualized by Pymol.

### 4.10. Evaluation of WLP-Derived Key Active Components Against MASLD via Cell Experiments

HepG2 cells were cultured in DMEM containing 10% fetal bovine serum and 1% penicillin-streptomycin and placed in a 37 °C, 5% CO_2_ cell incubator. HepG2 cells were treated with 1 mM free fatty acid (FFA; oleic acid: palmitic acid = 2:1) for 24 h to establish a steatosis model when the cells were in the logarithmic phase of growth. The HepG2 cells were treated with 5 and 20 μg/mL component group (CG; consisting of AF, AF-24, DMA, PA, AA-23A, DEO, PAA, HYD, OG, MET, AL-II and CA in the ratio 1:8.42:8.37:1.56:1.75:24.53:1.02:3.51:5.88:1.86:16.78:1.14; their concentration ratios were based on the ratio of chromatographic peak areas of the WLP-derived components in the serum), 10 and 40 μM of monomers (MET, DEO), and FC (150 μM) for 24 h. Finally, lipid accumulation in cells was observed through Oil Red O staining, and the intracellular levels of TC, TG, ALT, AST, GSH, SOD, IL-1β, IL-6 and TNF-α were detected.

### 4.11. Detection of NOS2 and PLA2G2A Expression by Western Blotting

Livers from rats and HepG2 cell samples were lysed using RIPA lysis buffer containing protease inhibitors. After centrifuging the suspension, the supernatant was collected. The protein concentration was determined using the BCA method. Equal amounts of protein extracts were mixed with loading buffer and denatured (100 °C, 10 min). Then, the protein samples were electrophoresed on a 10% SDS-PAGE gel and transferred to a polyvinylidene fluoride membrane, which was then placed in blocking solution (5% skimmed milk) for blocking for 1 h. The membrane was incubated with primary antibodies (anti-iNOS and anti-PLA2G2A) at 4 °C overnight, followed by incubation with Goat Anti-Rabbit secondary antibody at room temperature for 1 h. Finally, images were captured using a fully automatic chemiluminescence image analysis system (Tanon, Shanghai, China), and the results were analyzed using Image J software (Image J Software, version 1.54f, Bethesda, MD, USA).

### 4.12. Statistical Analysis

Statistical analyses were performed using GraphPad Prism 9.0 (GraphPad Software, version 9.5.1, San Diego, CA, USA), and the results were expressed as mean ± standard deviation. For comparisons between groups, one-way ANOVA or Wilcoxon rank-sum test was used, and *p* < 0.05 was considered statistically significant.

## 5. Conclusions

WLP improved liver tissue morphology and lipid accumulation in MASLD rats, as well as regulated hepatic lipid metabolism, oxidative stress, and inflammation. Its mechanism may be associated with WLP reversing the levels of 222 endogenous metabolites in MASLD rats, regulating 19 metabolic pathways, reducing the abundance of potential pathogenic bacteria *Romboutsia* and *Turicibacter*, and restoring the structure of gut microbiota. Additionally, 31 WLP-derived components were identified in rat serum. Thirteen of these were the key active components of WLP against MASLD, which can act on 11 targets and regulate 8 metabolic pathways and 20 differential metabolites. Additionally, DEO and MET downregulated the expression of NOS2 and PLA2G2A in cells, respectively, and alleviated hepatic steatosis.

## Figures and Tables

**Table 1:** OPLS-DA model parameters.

	Group	R^2^Y			Q^2^		
		Serum	Liver	Urine	Serum	Liver	Urine
ESI+	CON vs. MOD	0.995	0.996	0.998	0.774	0.847	0.965
	WLP vs. MOD	0.999	0.996	0.997	0.768	0.641	0.811
ESI−	CON vs. MOD	0.998	0.992	0.993	0.93	0.87	0.958
	WLP vs. MOD	0.999	0.998	0.998	0.894	0.688	0.85

CON, control group; MOD, model group; WLP, Wuling Powder group; ESI+, positive ion mode; ESI−, negative ion mode.

**Table 2:** WLP-derived components in serum of MASLD rats.

NO	RT (min)	[M + H] + ([M + Na]) + *m*/*z*	ESI − MSn (+) *m*/*z*	[M − H] − *m*/*z*	ESI − MSn (−) *m*/*z*	Predicted (*m*/*z*)	Measured (*m*/*z*)	Predicted Formula	Diff (ppm)	Assigned Identification	Type	Source
1	2.719	263.1678	203.1815, 163.0151, 145.1026, 117.0703	-	-	263.1657	263.1678	C_16_H_22_O_3_	7.98	8β-Methoxyatractylenolide I	Sesquiterpenoids	AMR
2	3.191	119.0346	119.0851	-	-	119.0339	119.0346	C_4_H_6_O_4_	5.88	Succinic acid	Carboxylic acids	PS, CR
3	3.504	149.0599	149.0231, 131.9504, 103.9433, 102.9259	-	-	149.0597	149.0599	C_9_H_8_O_2_	1.34	Cinnamic acid	Phenylpropanoic acid	CR
4	5.128	188.0707	170.0583, 118.0651	-	-	188.0706	188.0707	C_11_H_9_NO_2_	0.53	3-Indoleacrylic acid	Organic acids	AMR
5	5.233	217.1589	217.0221, 199.0294, 175.0312, 171.8645, 143.8713, 133.0469, 121.101, 119.0344			217.1587	217.1589	C_15_H_20_O	0.92	Atractylone	Sesquiterpenoids	AMR
6	6.141	389.2569	275.1801, 229.159	-	-	389.2534	389.2569	C_20_H_36_O_7_	8.99	teradecylcitric acid	Organic acids	AMR
7	7.383	475.3740	457.3148	-	-	475.3782	475.3740	C_30_H_50_O_4_	−8.84	11-deoxyalisol A	Triterpenoids	AR
8	7.645	455.3493	437.3066			455.352	455.3493	C_30_H_46_O_3_	−5.93	Dehydrotrametenolic acid	Triterpenoids	PA
9	7.891	529.3886	407.7912, 295.1999			529.3888	529.3886	C_33_H_52_O_5_	−0.38	Pachymic acid	Triterpenoids	PA
10	8.956	550.4046	455.8768, 419.9737			550.4095	550.4046	C_32_H_52_O_6_	−8.90	Alisol A 23-acetate	Triterpenoids	AR
11	10.545	261.1123	235.0274	-	-	261.1121	261.1123	C_15_H_32_O_3_	0.77	1,2,15-Pentadecanetriol	Fatty alcohol	AMR
12	12.892	163.0386	135.0403, 107.0502, 103.9498	-	-	163.039	163.0386	C_9_H_6_O_3_	−2.45	7-hydroxycoumarin	Coumarin	AMR
13	13.167	429.1731	295.2427, 163.1099			429.1755	429.1731	C_20_H_28_O_10_	−5.59	Cinnamylalcohol-6′-o-α-furanara-binose-O-β-glucopyranoside	Glycoside	CR
14	16.906	415.3217	157.0079, 129.0547, 105.07	-	-	415.3207	415.3217	C_27_H_42_O_3_	2.41	Diosgenin	Steroids	AMR
15	19.879	233.1535	233.1535, 189.0621, 159.0804, 145.1011, 133.1012, 131.0854, 105.0698	-	-	233.1536	233.1535	C_15_H_20_O_2_	−0.43	Atractylenolide II	Sesquiterpenoids	AMR, PS
16	20.811	203.1795	147.0451, 119.0624			203.1794	203.1795	C_15_H_22_	0.49	Alpha-Curcumene	Sesquiterpenoids	AMR
17	22.182	496.3416	478.3297, 419.2535, 184.2901, 104.1068			496.3421	496.3416	C_31_H_45_NO_4_	−1.01	7-[4-(11-hydroxy-undecyloxy)-phenyl]-7-pyridin-3-yl-hept-6-enoic acid ethyl ester	Organic acids	AR
18	22.453	507.3641	507.2712, 281.2471			507.368	507.3641	C_30_H_50_O_6_	−7.69	13β,17β-epoxyalisol A	Steroids	AR
19	26.015	233.1541	159.0804, 145.0641, 131.0854, 117.07, 115.0544	-	-	233.1542	233.1541	C_15_H_20_O_2_	−0.43	3β-Hydroxyatractylon	Sesquiterpenoids	AMR
20	27.201	453.3366	435.3331, 339.2755	-	-	453.3363	453.3366	C_30_H_44_O_3_	0.66	Dehydrotrametenonic acid	Triterpenoids	PA
21	29.613	455.3515	437.3306	-	-	455.352	455.3515	C_30_H_46_O_3_	−1.10	Alisol I	Triterpenoids	AR
22	31.210	189.1122	145.023, 127.054	-	-	189.1121	189.1122	C_9_H_16_O_4_	0.53	Azelaic acid	Carboxylic acids	CR
23	35.223	531.3683	435.2209	-	-	531.368	531.3683	C_32_H_50_O_6_	0.56	Alisol F 24-acetate	Triterpenoids	AR
24	35.275	531.3685	415.1964, 281.1909, 167.1442	-	-	531.3680	531.3685	C_32_H_50_O_6_	0.94	3−O−acetyl−16α,26−dihydroxytrametenolic acid	Triterpenoids	PA
25	4.297	-	-	167.0351	123.0055	167.035	167.0351	C_8_H_8_O_4_	0.60	Vanillic acid	Phenolic acids	CR
26	14.047			281.1398	281.2479, 191.8818	281.1395	281.1398	C_15_H_22_O_5_	1.07	Octyl gallate	Phenolic acids	AMR
27	18.713	-	-	487.3430	381.1656, 363.9795	487.3429	487.3430	C_30_H_48_O_5_	0.21	Alisol F	Triterpenoids	AR
28	18.713	-	-	487.3430	453.9094, 337.2613	487.3429	487.3430	C_30_H_48_O_5_	0.21	15,16-dihydroalisol A	Triterpenoids	AR
29	18.766			487.3432	451.3303	487.3429	487.3432	C_30_H_48_O_5_	0.62	3α,16α,25-Trihydroxylanosta-8,24-dien-21-oic acid	Triterpenoids	PA
30	25.159			497.3272	379.2705, 325.0071	497.3272	497.3272	C_31_H_46_O_5_	0.00	Poricoic acid A	Triterpenoids	PA
31	26.228	-	-	265.1444	265.147, 185.5014	265.1445	265.1444	C_15_H_22_O_4_	−0.38	6-hydroxy-3,3a-dihydro atractylenolide III	Sesquiterpenoids	AMR

AR, Alismatis Rhizoma; PA, Poria; PS, Polyporus; AMR, Atractylodis Macrocephalae Rhizoma; CR, Cinnamomi Ramulus.

**Table 3:** Docking information between key components and core targets.

Component	Target	Binding Energy (kcal/mol)	Amino Acid Residue
Alisol F	TKT	−7.6	HIS-54, ARG-57
Alisol F 24-acetate	NOS2	−8.8	TYR-150, LYS-191, and TRP-206
Dehydrotrametenolic acid	NOS2	−7	ARG-195, SER-153
Dehydrotrametenonic acid	NOS2	−8.4	SER-453, ARG-195
Dehydrotrametenonic acid	PLA2G4A	−6.8	TYR-96, ASP-40
Dehydrotrametenonic acid	MIF	−5.7	GLN-24, ASP-16
Pachymic acid	NOS2	−7.1	SER-453, GLU-450, SER-153, and ARG-195
Pachymic acid	PLA2G2A	−5.4	LYS-115, ASN-114
Alisol A 23-acetate	NOS2	−7.9	ARG-195, GLN-192, ASP-184, and SER-453
11-deoxyalisol A	NOS2	−8.4	ARG-195
Poricoic acid A	PLA2G4A	−7.3	ASP-40, TYR-96, ASN-95, VAL-97, and MET-98
7-hydroxycoumarin	MIF	−8.4	ILE-64, LYS-32, and ASN-97
7-hydroxycoumarin	MAOA	−8	VAL-244, ALA-44, and LEU-277
7-hydroxycoumarin	CYP1A2	−9	GLYn-6, ASP-313
7-hydroxycoumarin	ALDH2	−7.4	GLN-447, ARG -77, ARG -67, and ASP -239
7-hydroxycoumarin	ACHE	−8	GLU-202, SER-125, TYR-124
Octyl gallate	MIF	−6.6	PRO-1, LYS-32, and ILE-64
Octyl gallate	MAOA	−5.5	ASP-61, VAL-220, and GLM-225
Octyl gallate	ACHE	−10.2	SER-203, HIS-447, GLY-122, and TYR-124
Octyl gallate	MAOB	−10.7	THR-195
Octyl gallate	COMT	−7.7	CYS-119, ALA-117, ASP-191, and SER-122
8β-Methoxyatractylenolide I	PLA2G2A	−5.3	GLY-32
Atractylenolide II	MAOA	−9	ILE-180, ILEb-335, ILE-325, PHE-208, LEU-337, GLU-216, and PHE-352
Cinnamic acid	MAOB	−8.4	THR-196, THR-195

## Data Availability

The original contributions presented in the study are included in the article/Supplementary Material; further inquiries can be directed to the corresponding authors.

## Supplementary Materials

The following supporting information can be downloaded at https://www.mdpi.com/article/10.3390/ph19040557/s1, Figure S1: Quantity control of WLP extract; Table S1: Differential metabolites in rat serum; Table S2: Differential metabolites in rat liver; Table S3: Differential metabolites in rat urine.

## Abbreviations

AA-23A	alisol A 23-acetate
AF	alisol F
AF-24	alisol F 24-acetate
AL-II	atractylenolide II
ALT	alanine transaminase
AMR	atractylodis macrocephalae rhizoma
AR	Alismatis Rhizoma
AST	aspartate transaminase
BCA	bicinchoninic acid
CA	cinnamic acid
CG	component group
CON	control group
CR	Cinnamomi Ramulus
DEO	11-deoxyalisol A
DMA	dehydrotrametenolic acid
DMEM	Dulbecco’s modified eagle medium
ESI	electrospray ionization
FC	fenofibrate capsules group
FFA	fatty acid
GSH	glutathione
HE	hematoxylin-eosin
HDL-C	high density lipoprotein-C
HWLP	high-dose WLP group
HYD	7-hydroxycoumarin
IL-1β	interleukin 1β
IL-6	interleukin 6
LDL-C	low-density lipoprotein-C
LWLP	low-dose WLP group
MASLD	metabolic dysfunction-associated steatotic liver disease
MDA	malondialdehyde
MET	8β-Methoxyatractylenolide I
MOD	model group
NOS2	nitric oxide synthase 2
OG	octyl gallate
PLS-DA	orthogonal least partial squares discriminant analysis
OTUs	operational taxonomic units
PA	pachymic acid
PAA	poricoic acid A
PBS	phosphate-buffered saline
PC	phosphatidylcholine
PCA	principal component analysis
PCoA	principal coordinate analysis
PCR	polymerase chain reaction
PE	phosphatidylethanolamine
PM	poriae mushroom
PLA2G2A	phospholipase A2 group IIA
PS	Polyporus
QC	quality control
SOD	superoxide dismutase
TC	total cholesterol
TCM	traditional Chinese medicine
TG	triglyceride
TNF-α	tumor necrosis factor-α
VLDL	very low-density lipoprotein
WLP	Wuling powder
