# Supplementary material for: Unraveling the Mechanisms of Wuling Powder Against MASLD by Integrated Metabolomics–Gut Microbiota–Serum Pharmacochemistry
Source: Pharmaceuticals (Basel). 2026 Mar 31;19(4):557. doi: 10.3390/ph19040557 (PMC13119202; doi:10.3390/ph19040557)
Supplement: Supplementary file 1 [file pharmaceuticals-19-00557-s001.zip › pharmaceuticals-4216367-supplementary.pdf]

SUPPLEMENTARY MATERIAL

Unraveling the Mechanisms of Wuling Powder Against MASLD by Integrated Metabolomics–Gut Microbiota–Serum Pharmacochemistry

Huan Yang [1,2,†], Yan-Mei Tang [1,2,†], Shao-Cong Han [2,3], Peng-Quan Wang [1,2], Yu-Xuan Tao [1,2], Hui-Qiong Yang [1,2], Min Zhang [1,2], Min Li [1,2], Jie Yu [1,2,*] and Xing-Xin Yang [1,2,*]

[1] College of Pharmaceutical Science, Yunnan University of Chinese Medicine, 1076 Yuhua Road,
Kunming 650500, China; yanghuan_1205@163.com (H.Y.); m18488829594@163.com (Y.-M.T.); axero555@163.com (P.-Q.W.); ttaoyx@163.com (Y.-X.T.); 15752819630@163.com (H.-Q.Y.); 15187474645@163.com (M.Z.); lm08212000@163.com (M.L.)

[2] Yunnan Key Laboratory of Southern Medicine Utilization, 1076 Yuhua Road, Kunming 650500, China; hanshaocong_0225@163.com.

[3] The First Clinical College, Yunnan University of Chinese Medicine, 1076 Yuhua Road,
Kunming 650500, China

* Correspondence: yujie@ynucm.edu.cn (J.Y.); yxx78945@163.com (X.-X.Y.); Tel./Fax: +86-871-67496642 (J.Y.); +86-871-67496642 (X.-X.Y.)

† These authors contributed equally to this work.

Table of Contents

Supplementary methods

Supplementary Figures and Tables

1. **Fig. S1.** Quantity control of WLP extract.

2. **Table S1.** Differential metabolites in rat serum.

3. **Table S2.** Differential metabolites in rat liver.

4. **Table S3.** Differential metabolites in rat urine.

Supplementary methods

Quality control of WLP extract

The content of cinnamaldehyde was determined using an Agilent 1290 Infinity Ultra UPLC (Agilent Technologies, Santa Clara, CA, USA) to control the quality of WLP extract. Cinnamaldehyde and the freeze-dried powder of WLP extract were accurately weighed, and each was dissolved in methanol to prepare a 10 μg/mL solution and a 40 mg/mL solution, respectively. The solutions were subjected to ultrasonic treatment for 30 minutes, filtered through a 0.22 μm microporous membrane, and then analyzed by UPLC. Elution was performed using an Agilent ZORBAX SB-C18 chromatographic column (4.6×250 mm, 5 μm, Agilent Technologies, CA, USA) at a constant flow rate of 1.0 mL/min, with the column temperature maintained at 30 °C; the injection volume was 10 μL; the mobile phase consisted of acetonitrile (A) and 0.1% phosphoric acid aqueous solution (B). The gradient elution conditions were as follows: 0-10.0 min, 19% A；10.0-45.0 min, 30% A；45.0-70.0 min, 45% A；70.0-112.0 min, 70% A；112.0-115.0 min, 70% A. The detection wavelength was set at 280 nm.

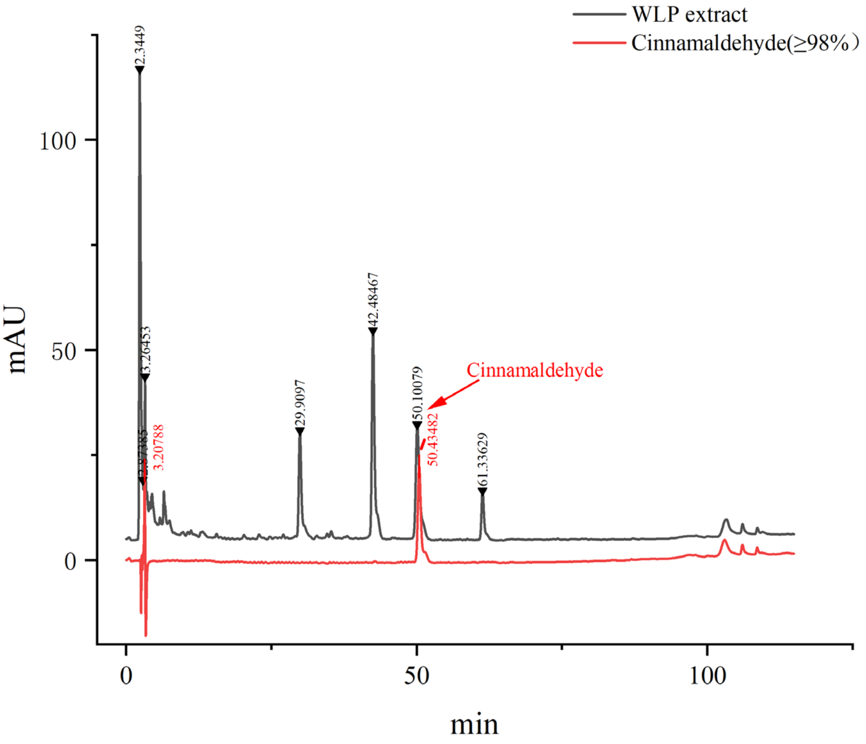

Figure. S1. **Quantity control of WLP extract.** The content of corresponding substances in the WLP extract was indicated by calculating based on the positions and peak areas of the standards.

Table S1. Differential metabolites in rat serum.

Retention time (min)	HMDB ID	m/z	Metabolite	Formula	MOD vs CON	WLP vs MOD
ESI+						
0.4591	HMDB0013751	137.0709	2-Hydroxypyridine	C_5H_5NO	up	down
3.5583	HMDB0256702	601.2667	Porfimer Sodium	$C_{68}H_{74}N_8O_{11}$	up	down
5.393	HMDB0000413	269.1358	3-Hydroxydodecanedioic acid	$C_{12}H_{22}O_5$	down	up
5.9414	HMDB0248844	534.2953	Mayzent	$C_{29}H_{35}F_3N_2O_3$	down	up
0.4744	HMDB0255498	331.1109	5'-N-Ethylcarboxamidoadenosine	$C_{12}H_{16}N_6O_4$	up	down
5.2695	HMDB0061643	269.1357	3-carboxy-4-methyl-5-pentyl-2-furanpropanoic acid	$C_{14}H_{20}O_5$	down	up
5.2695	HMDB0000453	229.1433	Epsilon-Caprolactone	$C_6H_{10}O_2$	down	up
5.7326	HMDB0249206	373.2735	Bile acid	$C_{24}H_{40}O_5$	down	up
0.8399	HMDB0242109	157.0971	N(5)-Acetylornithine	$C_7H_{14}N_2O_3$	down	up
5.8722	HMDB0011503	454.2927	PE(16:0/0:0)	$C_{21}H_{44}NO_7P$	down	up
7.8821	HMDB0062520	330.3364	3,7R,11R,15-tetramethyl-hexadecanoic acid	$C_{20}H_{40}O_2$	down	up

5.5323	HMDB0257199	443.2331	RHODAMINE 6G	$C_{28}H_{30}N_2O_3$	up	down
0.6217	HMDB0255276	137.0709	Methylene bisacrylamide	$C_7H_{10}N_2O_2$	up	down
0.3656	HMDB0000214	133.0972	Ornithine	$C_5H_{12}N_2O_2$	down	up
ESI-						
4.7057	HMDB0036274	790.3431	Quillaic acid 3-[galactosyl-(1->2)-[rhamnosyl-(1->3)]-glucuronide] 28-[xylosyl-(1->4)-rhamnosyl-(1->2)-[rhamnosyl-(1->3)]-4acetyl-fucosyl] ester	$C_{73}H_{114}O_{37}$	up	down
5.6632	HMDB0242008	505.3161	N-Docosahexaenoyl Arginine	$C_{28}H_{44}N_4O_3$	up	down
5.7032	HMDB0247856	233.0187	2,2,2-Trifluoro-N-(2-nitrophenyl) acetamide	$C_8H_5F_3N_2O_3$	down	up
5.7032	HMDB0000619	453.2860	Cholic Acid	$C_{24}H_{40}O_5$	down	up
7.8296	HMDB0013336	452.2785	3-Hydroxyhexadecanoylcarnitine	$C_{23}H_{45}NO_5$	down	up
5.7191	HMDB0298456	637.4325	DG(i-12:0/0:0/20:4(6E,8Z,11Z,13E)-2OH(5S,15S))	$C_{35}H_{60}O_7$	down	up
5.7032	HMDB0259922	231.0216	XANTHONE	$C_{13}H_8O_2$	down	up
4.9535	HMDB0060829	473.1454	Lacosamide-glucuronide	$C_{18}H_{24}N_2O_{10}$	down	up
4.1216	HMDB0000779	165.0549	3-Phenyllactic Acid	$C_9H_{10}O_3$	down	up

3.1719	HMDB0029200	273.0077	Ferulic acid 4-O-sulfate	$C_{10}H_{10}O_7S$	down	up
3.1401	HMDB0000714	178.0503	Hippuric Acid	$C_9H_9NO_3$	down	up
7.0767	HMDB0303085	453.2855	1-(4-O-beta-D-glucopyranosyl-3-methoxyphenyl)-3,5-dihydroxydecane	$C_{25}H_{42}O_7$	down	up
5.7032	HMDB0000505	407.2805	Allocholic acid	$C_{24}H_{40}O_5$	down	up
7.0687	HMDB0000865	407.2805	Hyocholic acid	$C_{24}H_{40}O_5$	down	up

Table S2. Differential metabolites in rat liver.

Retention time (min)	HMDB ID	m/z	Metabolite	Formula	MOD vs CON	WLP vs MOD
ESI+						
0.738	HMDB0003464	146.0924	4-Guanidinobutanoic Acid	$C_5H_{11}N_3O_2$	down	up
2.0562	HMDB0000875	138.0549	Trigonelline	$C_7H_7NO_2$	down	up
0.7457	HMDB0028810	263.1712	Glutaminylvaline	$C_{10}H_{19}N_3O_4$	down	up
ESI-						
2.9719	HMDB0256031	246.0442	P-Aminophenazone	$C_{12}H_9N_3O$	up	down
5.5721	HMDB0246603	257.0821	4,4'-Dihydroxystilbene	$C_{14}H_{12}O_2$	down	up

Table S3. Differential metabolites in rat urine.

Retention time (min)	HMDB ID	m/z	Metabolite	Formula	MOD Vs CON	WLP vs MOD
ESI+						
5.7433	-	413.2656	Pregnan-20-one, 17-(acetyloxy)-3-hydroxy-6-methyl-, (3b,5b,6a)-	$C_{24}H_{38}O_4$	up	down
4.2150	HMDB0000764	133.0647	Hydrocinnamic acid	$C_9H_{10}O_2$	down	up
3.8022	-	447.0917	Apigenin-7-Glucuronide	$C_{21}H_{18}O_{11}$	down	up
3.2573	-	461.1073	Oroxindin	$C_{22}H_{20}O_{11}$	down	up
3.1955	HMDB0041717	431.0968	Daidzein 4'-O-glucuronide	$C_{21}H_{18}O_{10}$	down	up
1.4418	HMDB0029075	230.1134	Threoninyl-Gamma-glutamate	$C_9H_{17}N_3O_5$	down	up
3.3117	HMDB0257775	297.144	3a-Methyl-2,3,4,5,5a,10,10a,10b-octahydro-1H-cyclopenta[a]fluorene-2,3,7-triol	$C_{17}H_{22}O_3$	down	up
2.5293	HMDB0241918	358.1129	N-Arachidonoyl Glycine	$C_{15}H_{19}NO_9$	down	up
0.4269	HMDB0001432	131.1291	Agmatine	$C_5H_{14}N_4$	down	up
4.6283	HMDB0041732	436.1598	Equol 7-O-glucuronide	$C_{21}H_{22}O_9$	down	up

1.1457	HMDB0060477	200.1028	Gamma-Glutamyl-beta-aminopropiononitrile	$C_8H_{13}N_3O_3$	down	up
1.4105	HMDB0011655	289.1501	2-(3-Carboxy-3-aminopropyl)-L-histidine	$C_{10}H_{16}N_4O_4$	down	up
2.0731	HMDB0301846	264.1591	Lycopsamine	$C_{15}H_{25}NO_5$	down	up
2.3276	HMDB0062645	282.1443	N(2)-phenylacetyl-L-glutaminate	$C_{13}H_{16}N_2O_4$	down	up
2.7609	HMDB0245137	146.0922	2-Guanidinobutanoic acid	$C_5H_{11}N_3O_2$	down	up
2.8775	HMDB0006050	182.0811	DL-o-Tyrosine	$C_9H_{11}NO_3$	down	up
2.8775	HMDB0028764	284.1058	Aspartyl-Tryptophan	$C_{15}H_{17}N_3O_5$	down	up
3.1410	HMDB0035140	265.1431	(+)-Abscisic Acid	$C_{15}H_{20}O_4$	down	up
3.1955	HMDB0003312	255.0648	Daidzein	$C_{15}H_{10}O_4$	down	up
3.2573	HMDB0002338	285.0754	Biochanin A	$C_{16}H_{12}O_5$	down	up
3.2651	HMDB0001067	322.1216	N-Acetyl-1-aspartylglutamic acid	$C_{11}H_{16}N_2O_8$	up	down
3.3272	HMDB0000500	121.0285	4-Hydroxybenzoic Acid	$C_7H_6O_3$	down	up
3.4518	HMDB0037560	316.175	3,7,8,15-Scirpenetetrol	$C_{15}H_{22}O_6$	down	up
3.7555	HMDB0030808	257.0805	Pinocembrin	$C_{15}H_{12}O_4$	down	up
3.7555	HMDB0037338	493.145	Americanin B	$C_{27}H_{24}O_9$	down	up
3.8100	HMDB0303804	252.1593	7-Epijasmonic acid	$C_{12}H_{18}O_3$	down	up
3.9267	HMDB0060289	340.132	Quinoline-4,8-diol	$C_9H_7NO_2$	up	down

4.0358	HMDB0002271	158.0923	Imidazolepropionic acid	$C_6H_8N_2O_2$	down	up
4.0513	HMDB0030179	170.0599	Edulitine	$C_{11}H_{11}NO_3$	down	up
4.7609	HMDB0013327	374.2534	Dodecanedioylcarnitine	$C_{19}H_{35}NO_6$	down	up
4.9171	HMDB0240259	595.3482	Stercobilin	$C_{33}H_{46}N_4O_6$	up	down
5.1589	HMDB0243583	406.0951	2-(N-(7-Nitrobenz-2-oxa-1,3-diazol-4-yl)amino)-2-deoxyglucose	$C_{12}H_{14}N_4O_8$	down	up
5.2603	HMDB0033917	267.1951	4-Hydroxy-3-methoxy-2,10-bisaboladien-9-one	$C_{16}H_{26}O_3$	down	up
5.3226	HMDB0036692	345.1663	Heliangin	$C_{20}H_{26}O_6$	down	up
2.5137	HMDB0033092	185.0807	3-(1-Hydroxymethyl-1-propenyl)pentanedioic acid	$C_9H_{14}O_5$	down	up
7.9632	HMDB0041731	436.1597	Equol 4'-O-glucuronide	$C_{21}H_{22}O_9$	down	up
5.7511	HMDB0304813	252.2319	Xestoaminol C	$C_{14}H_{31}NO$	up	down
5.5568	HMDB0012109	411.2349	5,6-Dihydroxyprostaglandin F1a	$C_{20}H_{36}O_7$	down	up
5.1667	HMDB0241082	326.1958	Dec-4-enedioylcarnitine	$C_{17}H_{29}NO_6$	down	up
4.9171	HMDB0037316	257.0805	Isoliquiritigenin	$C_{15}H_{12}O_4$	down	up
4.8389	HMDB0030622	319.1178	(R)-Oxypeucedanin	$C_{16}H_{14}O_5$	down	up

4.8233	HMDB0031711	272.2581	15-Hexadecanolide	$C_{16}H_{30}O_2$	up	down
0.4731	HMDB0253198	176.0106	Homarine	$C_7H_7NO_2$	down	up
4.5503	HMDB0041044	374.2532	5,7-Megastigmadien-9-ol glucoside	$C_{19}H_{32}O_6$	down	up
4.4957	HMDB0030206	762.387	Jubanine B	$C_{43}H_{47}N_5O_6$	up	down
4.4801	-	278.1599	Dihydro-3-hydroxy-4,4-dimethyl-2(3H)-Furanone	$C_6H_{10}O_3$	down	up
4.4410	HMDB0037906	281.149	Hernandulcin	$C_{15}H_{24}O_2$	down	up
4.2306	HMDB0029012	271.1399	Prolyl-Asparagine	$C_9H_{15}N_3O_4$	down	up
4.2150	HMDB0246092	308.104	3'-DEOXY-3'-FLUOROTHYMIDINE	$C_{10}H_{13}FN_2O_4$	down	up
0.6025	HMDB0015095	271.1397	Pindolol	$C_{14}H_{20}N_2O_2$	down	up
4.0513	HMDB0036607	252.1592	(S)-Pterosin P	$C_{14}H_{18}O_3$	down	up
4.0513	HMDB0001190	142.0649	Indoleacetaldehyde	$C_{10}H_9NO$	down	up
3.8877	HMDB0000866	188.0704	N-Acetyltyrosine	$C_{11}H_{13}NO_4$	down	up
3.8334	-	134.0963	D-Cathine	$C_9H_{13}NO$	down	up
3.7243	HMDB0000624	287.1486	D-Leucic acid	$C_6H_{12}O_3$	down	up
3.6855	HMDB0254850	241.1432	2,3-Dihydroxypropyl octanoate	$C_{11}H_{22}O_4$	down	up
3.6075	HMDB0060564	219.1014	Carboxyibuprofen	$C_{13}H_{16}O_4$	down	up

3.4362	HMDB0246408	324.1913	4-Deoxypyridoxine	$C_8H_{11}NO_2$	down	up
3.4206	HMDB0000679	379.2334	Homocitrulline	$C_7H_{15}N_3O_3$	up	down
3.4050	MJDBOTE0001787	165.0545	3-Coumaric Acid	$C_9H_8O_3$	down	up
3.3972	HMDB0001218	147.0439	Coumarin	$C_9H_6O_2$	down	up
3.3816	HMDB0029657	322.1313	2,4'-Dihydroxyacetophenone	$C_8H_8O_3$	up	down
0.8032	HMDB0031842	146.0922	4-Guanidinobutanoic acid	$C_5H_{11}N_3O_2$	down	up
3.1721	HMDB0028830	298.1214	Glutamyltryptophan	$C_{16}H_{19}N_3O_5$	down	up
0.6025	HMDB0000904	158.0922	Citrulline	$C_6H_{13}N_3O_3$	down	up
2.9931	HMDB0247174	237.123	Metyrosine	$C_{10}H_{13}NO_3$	down	up
2.9854	HMDB0015451	148.1119	Ephedrine	$C_{10}H_{15}NO$	down	up
2.9778	HMDB0244101	159.0439	1,2-Naphthoquinone	$C_{10}H_6O_2$	down	up
1.0212	HMDB0250742	158.0923	D-Citrulline	$C_6H_{13}N_3O_3$	down	up
2.6835	HMDB0035665	217.097	L-1,2,3,4-Tetrahydro-beta-carboline-3-carboxylic acid	$C_{12}H_{12}N_2O_2$	down	up
2.6757	HMDB0041802	144.0806	2-Naphthylamine	$C_{10}H_9N$	down	up
2.5137	HMDB0252174	197.1282	Fasoracetam	$C_{10}H_{16}N_2O_2$	down	up
2.4284	HMDB0028959	285.1917	Lysylproline	$C_{11}H_{21}N_3O_3$	down	up

2.1196	HMDB0060336	235.1438	1,4'-Bipiperidine-1'-carboxylic acid	$C_{11}H_{20}N_2O_2$	down	up
1.8008	HMDB0255802	253.1291	4-Amino-1-[5-(hydroxymethyl)oxolan-2-yl]pyrimidin-2-one	$C_9H_{13}N_3O_3$	down	up
3.4284	HMDB0000735	210.0759	Hydroxyphenylacetylglycine	$C_{10}H_{11}NO_4$	down	up
1.3951	HMDB0004350	163.1228	Anabasine	$C_{10}H_{14}N_2$	down	up
1.3717	HMDB0257731	290.1341	4-Amino-1-[(2R,5R)-3,4-dihydroxy-5-(hydroxymethyl)oxolan-2-yl]-5-methylpyrimidin-2-one	$C_{10}H_{15}N_3O_5$	down	up
1.1145	HMDB0246884	332.1336	5'-O-beta-D-Glucosylpyridoxine	$C_{14}H_{21}NO_8$	down	up
1.0833	HMDB0006488	138.0548	N-Acetyl-L-glutamate 5-semialdehyde	$C_7H_{11}NO_4$	down	up
0.5644	HMDB0003148	176.1028	Argininic acid	$C_6H_{13}N_3O_3$	down	up
0.5030	HMDB0012234	122.0712	Histidinal	$C_6H_9N_3O$	down	up
4.1759	HMDB0257722	449.1074	(2S,3S,4S,5R)-6-[4-(5,7-Dihydroxy-4-oxo-2,3-dihydrochromen-2-yl)phenoxy]-3,4,5-trihydroxyoxane-2-carboxylic acid	$C_{21}H_{20}O_{11}$	down	up
4.2228	HMDB0041740	461.1073	Glycitein 4'-O-glucuronide	$C_{22}H_{20}O_{11}$	down	up
0.6873	HMDB0004992	138.0548	P-Aminobenzoic acid	$C_7H_7NO_2$	down	up

ESI-

0.4813	HMDB0033626	315.0143	D-Erythroascorbic acid 1'-a-D-xylopyranoside	$C_{10}H_{14}O_9$	up	down
0.4813	HMDB0011676	355.0884	D-Xylono-1,5-lactone	$C_5H_8O_5$	up	down
0.5210	HMDB0000150	223.0454	Gluconolactone	$C_6H_{10}O_6$	down	up
0.6395	HMDB0251034	245.1142	Descyclopropyl Abacavir	$C_{11}H_{14}N_6O$	down	up
0.8447	HMDB0258911	285.049	TETRAHYDROURIDINE	$C_9H_{16}N_2O_6$	up	down
1.0031	HMDB0248966	292.036	Thiobencarb	$C_{12}H_{16}ClNOS$	down	up
1.3684	HMDB0029559	269.1257	(4-Hydroxybenzoyl)choline	$C_{12}H_{18}NO_3+$	down	up
1.8948	HMDB0015066	463.113	Methacycline	$C_{22}H_{22}N_2O_8$	up	down
2.1335	HMDB0124993	232.976	Protocatechuic acid 3-O-sulfate	$C_7H_6O_7S$	down	up
2.1655	HMDB0059724	188.9857	Pyrocatechol sulfate	$C_6H_6O_5S$	down	up
2.2211	HMDB0242531	323.0377	(2R)-2-Acetamido-3-[[(2R)-2-acetamido-2-carboxyethyl]disulfanyl]propanoic acid	$C_{10}H_{16}N_2O_6S_2$	down	up
2.2848	HMDB0002432	329.0517	Sumiki's acid	$C_6H_6O_4$	down	up
2.3645	-	323.0376	(2R,2'R)-3,3'-disulfanediylbis(2-acetamidopropanoic acid)	$C_{10}H_{16}N_2O_6S_2$	down	up
2.4680	HMDB0060804	203.9968	8-Hydroxythioguanine	$C_5H_5N_5OS$	down	up

2.4760	HMDB0304122	152.0106	3-dehydroshikimate	$C_7H_7O_5$-	down	up
2.4838	HMDB0140929	246.9917	4-methoxy-3-(sulfooxy)benzoic acid	$C_8H_8O_7S$	down	up
2.6025	HMDB0006331	343.0673	Cis,cis-Muconic acid	$C_6H_6O_4$	down	up
2.6817	HMDB0010350	343.1036	2-Phenylethanol glucuronide	$C_{14}H_{18}O_7$	down	up
2.8483	-	154.0137	Citrazinic Acid	$C_6H_5NO_4$	down	up
2.9672	HMDB0246781	205.0714	5-Dihydroxyhept-6-enoic acid	$C_7H_{12}O_4$	down	up
3.0783	HMDB0125166	242.9968	P-Coumaric acid sulfate	$C_9H_8O_6S$	down	up
3.0783	HMDB0240477	399.0931	5-Caffeoylquinic acid	$C_{16}H_{18}O_9$	down	up
3.1408	HMDB0029200	273.0076	Ferulic acid 4-O-sulfate	$C_{10}H_{10}O_7S$	down	up
3.1876	HMDB0037552	323.1168	4-Hydroxy-3-(2-hydroxyethyl)acetophenone 4-glucoside	$C_{16}H_{22}O_8$	down	up
3.3062	HMDB0252508	263.0231	[(2S,3S,4S,5S,6R)-4,5-Dihydroxy-2,6-dimethyloxan-3-yl] hydrogen sulfate	$C_7H_{14}O_7S$	down	up
3.4011	HMDB0041707	355.0674	Caffeic acid 4-O-glucuronide	$C_{15}H_{16}O_{10}$	down	up
3.4249	HMDB0001856	153.0184	Protocatechuic Acid	$C_7H_6O_4$	down	up
3.4722	HMDB0015350	313.0567	Dantrolene	$C_{14}H_{10}N_4O_5$	down	up
3.6221	HMDB0002428	165.0185	Terephthalic Acid	$C_8H_6O_4$	down	up

3.8118	HMDB0060933	399.1662	Propofol glucuronide	$C_{18}H_{26}O_7$	down	up
3.8593	HMDB0000840	194.0453	Salicyluric Acid	$C_9H_9NO_4$	down	up
3.8990	HMDB0251822	493.0789	Emtricitabine	$C_8H_{10}FN_3O_3S$	up	down
4.1207	HMDB0000779	147.0442	3-Phenyllactic Acid	$C_9H_{10}O_3$	down	up
4.3030	HMDB0035214	269.0456	Emodin	$C_{15}H_{10}O_5$	down	up
4.3189	HMDB0015182	307.1303	Abacavir	$C_{14}H_{18}N_6O$	down	up
4.4141	HMDB0001954	205.1076	3-Hydroxyoctanoic acid	$C_8H_{16}O_3$	down	up
4.4856	HMDB0243806	489.228	1-[[4-(Dimethylamino)-3-methylphenyl]methyl]-5-(2,2-diphenylacetyl)-6,7-dihydro-4H-imidazo[4,5-c]pyridine-6-carboxylic acid	$C_{31}H_{32}N_4O_3$	up	down
4.5173	HMDB0250224	299.1137	4,5-Dihydro-6-(4-(imidazol-1-yl)phenyl)-5-methyl-3(2H)-pyridazinone	$C_{14}H_{14}N_4O$	down	up
4.6282	HMDB0303245	205.1077	Isovaleraldehyde glyceryl acetal	$C_8H_{16}O_3$	down	up
4.7232	HMDB0245694	313.0931	4-O,6-O-Benzylidene-alpha-D-glucopyranose	$C_{13}H_{16}O_6$	down	up
4.9451	HMDB0060829	473.1455	Lacosamide-glucuronide	$C_{18}H_{24}N_2O_{10}$	down	up
4.9609	HMDB0038787	469.1659	Melleolide B	$C_{24}H_{32}O_7$	down	up

5.1907	HMDB0248946	317.0699	Bendazac	$C_{16}H_{14}N_2O_3$	up	up
5.5394	HMDB0253140	353.1245	Hexahydrophthalic anhydride	$C_8H_{10}O_3$	down	up
5.9191	HMDB0304923	218.9965	2-Methoxyhydroquinone sulfate	$C_7H_8O_6S$	down	up
6.0935	HMDB0303951	285.0618	(1R,6R)-6-hydroxy-2-succinylcyclohexa-2,4-diene-1-carboxylate	$C_{11}H_{12}O_6$	down	up
7.7932	HMDB0253761	379.0915	Kaltostat	$C_{14}H_{22}O_{13}$	down	up
2.9354	HMDB0039952	415.1226	1-(3-Methyl-2-butenoyl)-6-apiosylglucose	$C_{16}H_{26}O_{11}$	down	up
5.4525	HMDB0013912	327.1086	2-Hydroxynevirapine	$C_{15}H_{14}N_4O_2$	down	up
5.3891	HMDB0060582	433.1147	Benazeprilat	$C_{22}H_{24}N_2O_5$	down	up
5.3654	HMDB0302686	327.1087	Anisatin	$C_{15}H_{20}O_8$	down	up
3.1177	HMDB0304310	356.0988	Cyclo-dopa 5-O-glucoside	$C_{15}H_{19}NO_9$	down	up
5.1589	HMDB0039723	371.0814	3-O-alpha-D-Glucopyranuronosyl-D-xylose	$C_{11}H_{18}O_{11}$	down	up
5.1509	HMDB0304482	369.1193	Secologanin	$C_{17}H_{24}O_{10}$	down	up
4.9925	HMDB0304081	163.0392	Phenylpyruvic Acid	$C_9H_8O_3$	down	up
4.9766	HMDB0030598	419.1358	(-)-Wikstromol	$C_{20}H_{22}O_7$	down	up

4.6362	HMDB0247885	477.1769	3-[2-[[(1S,2R,3S)-3-[4-(Pentylcarbamoyl)-1,3-oxazol-2-yl]-7-oxabicyclo[2.2.1]heptan-2-yl]methyl]phenyl]propanoic acid	$C_{25}H_{32}N_2O_5$	down	up
4.6202	-	447.0936	Naringenin-7-O-beta-D-Glucuronide	$C_{21}H_{20}O_{11}$	down	up
4.5728	HMDB0059980	369.1191	4-Hydroxy-5-(phenyl)-valeric acid-O-glucuronide	$C_{17}H_{22}O_9$	down	up
4.4777	HMDB0041274	415.1613	Phenylethyl primeveroside	$C_{19}H_{28}O_{10}$	down	up
4.3347	HMDB0258290	265.1195	Siguazodan	$C_{14}H_{16}N_6O$	down	up
4.3189	HMDB0002302	234.077	Indole-3-propionic acid	$C_{11}H_{11}NO_2$	down	up
4.2317	HMDB0060653	289.0389	3-Hydroxycarbamazepine	$C_{15}H_{12}N_2O_2$	down	up
4.2237	HMDB0014703	489.1405	Methotrexate	$C_{20}H_{22}N_8O_5$	down	up
4.1761	HMDB0040797	399.1298	Methyl helianthenoate F glucoside	$C_{17}H_{22}O_8$	down	up
4.1286	HMDB0001713	163.0393	M-Coumaric acid	$C_9H_8O_3$	down	up
3.5591	HMDB0041724	275.0232	Dihydroferulic acid 4-O-sulfate	$C_{10}H_{12}O_7S$	down	up
4.0651	HMDB0031511	115.0753	Diacetone Alcohol	$C_6H_{12}O_2$	down	up
3.8751	HMDB0060354	295.1298	2-Polyprenyl-6-methoxy-1,4-benzoquinone	$C_{17}H_{22}O_3$	down	up
3.7801	HMDB0061112	239.0923	CMPF	$C_{12}H_{16}O_5$	down	up

3.7566	HMDB0247290	253.0506	7,4'-Dihydroxyflavone	$C_{15}H_{10}O_4$	down	up
3.7486	HMDB0037408	617.0972	Neocarlinoside	$C_{26}H_{28}O_{15}$	down	up
3.6537	HMDB0034863	371.134	4-(4-Hydroxyphenyl)-2-butanone glucoside	$C_{16}H_{22}O_7$	down	up
3.7566	-	429.0828	Chrysin-7-O-Glucuronide	$C_{21}H_{18}O_{10}$	down	up
3.4881	HMDB0041222	457.1541	A-L-Fucopyranosyl-(1->2)-b-D-galactopyranosyl-(1->2)-D-xylose	$C_{17}H_{30}O_{14}$	down	up
3.3931	HMDB0301685	621.1099	Apigenin 7-O-diglucuronide	$C_{27}H_{26}O_{17}$	down	up
3.3931	HMDB0062407	210.0768	5-(3-Pyridyl)-2-hydroxytetrahydrofuran	$C_9H_{11}NO_2$	down	up
3.1641	HMDB0041709	355.0673	Caffeoyl C1-glucuronide	$C_{15}H_{16}O_{10}$	down	up
3.1408	HMDB0034172	133.0284	Methyl 2-hydroxybenzoate	$C_8H_8O_3$	down	up
3.0703	HMDB0004072	119.0491	4-Vinylphenol	C_8H_8O	down	up
3.0544	HMDB0242135	240.9811	2H-1-Benzopyran-2-one, 7-(sulfooxy)-	$C_9H_6O_6S$	down	up
3.0068	HMDB0060566	303.018	Carbamazepine-O-quinone	$C_{15}H_{10}N_2O_3$	down	up
3.0068	HMDB0059999	153.0184	Gentisic Acid	$C_7H_6O_4$	down	up
2.9354	HMDB0041749	369.0827	Isoferuloyl C1-glucuronide	$C_{16}H_{18}O_{10}$	down	up
2.8799	HMDB0249757	498.0714	Cefcapene	$C_{17}H_{19}N_5O_6S_2$	down	up
2.8720	HMDB0011719	261.0075	Homovanillic acid sulfate	$C_9H_{10}O_7S$	down	up

2.7611	HMDB0301952	401.109	1-O-Feruloyl-beta-D-glucose	$C_{16}H_{20}O_9$	down	up
2.4918	HMDB0001336	167.0341	3,4-dihydroxyphenylacetic Acid	$C_8H_8O_4$	down	up
2.4123	HMDB0304906	277.0024	4-Hydroxyphenylacetic acid sulfate	$C_8H_8O_6S$	down	up
2.4123	HMDB0002085	197.045	Syringic Acid	$C_9H_{10}O_5$	down	up
2.3485	HMDB0010324	343.0674	Benzoyl glucuronide (Benzoic acid)	$C_{13}H_{14}O_8$	down	up
2.3166	HMDB0302044	343.1036	Chakanoside I	$C_{14}H_{18}O_7$	down	up
2.2848	HMDB0060568	307.0495	10,11-Dihydroxycarbamazepine	$C_{15}H_{14}N_2O_3$	down	up
2.2131	HMDB0259812	304.0134	Vinclozolin M2	$C_{11}H_{11}Cl_2NO_2$	down	up
2.1655	HMDB0031303	109.0284	Osmundalactone	$C_6H_8O_3$	down	up
2.1015	HMDB0012247	168.0294	L-2,3-Dihydrodipicolinate	$C_7H_7NO_4$	down	up
4.3030	HMDB0041832	445.0777	Baicalin	$C_{21}H_{18}O_{11}$	down	up
2.0777	HMDB0246707	245.0601	1,3-Diethyl-6-hydroxy-2-sulfanylidenepyrimidin-4-one	$C_8H_{12}N_2O_2S$	down	up
1.8392	HMDB0060017	301.0567	Pyrogallol-2-O-glucuronide	$C_{12}H_{14}O_9$	down	up
4.4062	HMDB0037254	253.0505	Garbanzol	$C_{15}H_{12}O_5$	down	up
1.0031	HMDB0001254	258.0385	Glucosamine 6-phosphate	$C_6H_{14}NO_8P$	up	down
0.6001	HMDB0002022	285.0493	Glycineamideribotide	$C_7H_{15}N_2O_8P$	up	down

4.7470	HMDB0041759	447.0936	Naringenin 4'-O-glucuronide	$C_{21}H_{20}O_{11}$	down	up
5.2146	HMDB0036773	263.129	Tavulin	$C_{15}H_{20}O_4$	down	up
3.6299	HMDB0060651	289.0389	2-Hydroxycarbamazepine	$C_{15}H_{12}N_2O_2$	down	up
3.0703	HMDB0002641	163.0393	2-Hydroxycinnamic Acid	$C_9H_8O_3$	down	up
4.1920	HMDB0034913	325.0938	Imazamethabenz-methyl	$C_{16}H_{20}N_2O_3$	down	up
